# Supplementary material for: Appropriate complementary feeding practices and associated factors among mothers with infants aged 6–8 months in West Gojjam Zone, Northwest Ethiopia: A mixed methods study
Source: PLoS One. 2025 Oct 16;20(10):e0334740. doi: 10.1371/journal.pone.0334740 (PMC12530518; doi:10.1371/journal.pone.0334740)
Supplement: S1 Appendix — (DOCX) [file pone.0334740.s001.docx]

**English version:** Interview guides to assess barriers and enablers of complementary feeding practices among mothers for their children

**A) In-depth Interview guide**

| **Participant’s background information** | | | | |
| --- | --- | --- | --- | --- |
| **Participant’ s code** | **Age** | **Level of education** | **Marital status** | **Number of children** |
| Mother |  |  |  |  |

1. Knowledge about complementary feeding practices (CFP) (probe: What is child complementary feeding mean? (Probe: Why complementary foods (CF) are needed in addition to breast milk? What is the amount of CF a child feed at a time? Any influence regarding CF?
2. What is an appropriate complementary feeding (ACF) mean**?** (Probe: What are the ingredients of porridge? What are the variety of CF a child feed? When you can say complementary feeding is appropriate? (continuing breastfeeding, dietary diversity, meal frequency, and animal source food (milk, egg, flesh food consumption).
3. How do you describe the challenges of ACF practices for a child below 2 years?

Probe:

- Preparing CF from variety food sources (in terms of cost, availability of food-stuffs & time)
- How do you describe the usual/cultural child feeding practices in your locality? (Probe: Any food taboo for this age group? Can you give example of food taboo? Why this can be done?)
- How do you think about feeding animal source foods (ASF) for a child below 2 years? **(**What are the benefits?)
- What do you think about providing ASF during fasting periods?
- What are the persons’ thought (family, grandmother/father, or community) about ACFP?
- What is your experience of complementary feeding practices to your child? (Probe: Please tell me your thought of preparing and feeding CF! Why not you practice CF appropriately? (in terms of the availability, cost, time, or working condition).

1. What are the things that make it possible for children to eat balanced diet? (Probe: How do you explain community and healthcare system support related to CF practices?)

- If anything, else regarding this issue, please describe it in detail?

Thank you for your time and cooperation!

**አማርኛ ቅጂ፡-** እናቶች ልጆቻቸውን የተመጣጠነ ተጨማሪ ምግብ ለመመገብ የማያስችሉ ተግዳሮቶችን እና የተመጣጠነ ምግብ ለመመገብ የሚያስችሉ ምቹ ሁኔታዎችን ለመገምገም የተዘጋጀ ምሪ ምጠይቅ።

ህ) **ኢንዴብዝ ኢንተርቪው** **(In-depth Interview guide)**

| **በቃለ መጠይቁ የተሳተፉ እናቶች አጭር ምረጃ** | | | | |
| --- | --- | --- | --- | --- |
| **መለያ (ምልክት)** | **እድሜ** | **የት/ርት ደረጃ** | **የጋብቻ ሁኔታ** | **የልጆች ብዛት** |
| **እናት** |  |  |  |  |

1. ስለ ተጨማሪ ምግብ አመጋገብ ልምድ እውቀት በተመለከተ (ምርመራ፡- የህጻን ተጨማሪ ምግብ ማለት ምን ማለት ነው? ከጡት ወተት በተጨማሪ ለህጻኑ ተጨማሪ ምግቦች ለምን ያስፈልጋሉ?) አንድ ህፃን በአንድ ጊዜ የሚመገበው የምግብ መጠን ምን ያክል ነው? በተጨማሪ ምግብ አመጋገብ በተመለከተ ምን ምን ተጽእኖ አለ?
2. የተመጣጣነ ተጨማሪ ምግብ ማለት ምን ማለት ነው? (ምርመራ፡- ገንፎ ከምን አይነት የእህል ስብጥር ሊዘጋጅ ይችላል? የተለያዩ የህፃናት የምግብ አይነቶች ስንል ምን ማለታችን ነው? ህጻኑ ተገቢ የሆነ ተጨማሪ ምግብ እየተመገበ ነው የሚሉት ምን ምን ምግቦችን ሲበላ ነው? (ምርመራ፡- የጡት ወተት ማጥባት መቀጠል፣ በእድሜው ልክ የተለያአዩ የምግብ አይነቶችን መመገብ ፣ በቀን ወሰጥ ምግብ ደጋግሞ መመገብ፣ እና የእንስሳት ምንጭ ምግቦች (ወተት፣ እንቁላል፣ የሥጋ ምግብ)።
3. ከ 2 ዓመት በታች ያሉ ህፃናትን ተመጣጣኝ ተጨማሪ ምግቦችን ለመመገብ የማያስችሉ ተግዳሮቶችን እንዴት ይገልጿችዋል?

**ምርመራ፡-**

- ከተለያዩ የምግብ ምንጮች ተጨማሪ ምግብ ማዘጋጀት (ምርመራ፡- ከዋጋ አንጻር፣ ምግብ ለምስራት ከሚያገለግሉ አቅርቦቶች አንጻር (እህል፣ ጥራጥሬ፣ ወተት፣ ስጋ ፣ እንቁላል፣ በቫይታሚን-ኤ የበለፀጉ ፍራፍሬዎች እና አትክልቶች)።
- በአካባቢዎ ያለውን የተለመደ/ባህላዊ የህፃናት አመጋገብ ሁኔታ እንዴት ይገልጹታል? (ምርመራ፡- በዚህ እድሜ ውስጥ ላሉ ህጻናት የሚከለከሉ ምግቦች ይኖራሉ? የሚከለከሉ ምግቦች ካሉ ምሳሌ ሊጠቅሱልኝ ይችላሉ? እንዲህ ለምን ያደርጋሉ? ቢያአብራሩልኝ?)
- ከ **2** ዓመት በታች ላሉ ህፃናት የእንስሳት ምንጭ ምግቦችን በመመገብ ዙሪያ ምን ያስባሉ? (ምርመራ፡- በዚህ እድሜ ውስጥ ያሉ ህጻናት መብላት ያለባቸው የእንስሳት ምንጭ የሆኑ ምግቦች ምን ምን ናችው? ጥቅማቸውስ ምን ሊሆን ይችላል?)
- በጾም ወቅት የእንስሳት ምንጭ የሆኑ ምግቦችን ለህጻናት አዘጋጅቶ በመመገብ ዙሪያ ምን ይላሉ?
- ሰዎች ​​(ቤተሰብ፣ አያት/አባት፣ የአካባቢ ሰዎች) በሀጻናት የተመጣጠነ ተጨማሪ ምግብ አመጋገብ ዙሪያ ምን ዓይነት አመለካከት/አስተሳሰብ/ አላቸው?
- ለልጅዎ የሚያደርጉትን የተጨማሪ ምግብ አመጋገብ ልምድዎን ቢያብራሩለኝ? (ምርመራ፡- ተጨማሪ ምግቦችን የማዘጋጀት እና የመመገብ ሃሳብዎን/አመለካከትዎን ቢያብራሩርልኝ።
- ተመጣጣኝ የሆነ ተጨማሪ ምግብ አምጋገብ ዘዴን ለምን በአግባቡ መከተል አይችሉም? (ምርመራ፡- ምግብ ለምስራት ከሚያገለግሉ አቅርቦቶች አንጻር (እህል፣ ጥራጥሬ፣ ወተት፣ ስጋ ፣ እንቁላል፣ በቫይታሚን-ኤ የበለፀጉ ፍራፍሬዎች እና አትክልቶች) እንዲሁም ከዋጋ፣ ጊዜ፣ የስራ ሁኔታ አንጻር ቢያብራሩልኝ) ።

1. ህጻናትን የተመጣጠነ ምግብ መመገብ የሚያስችሉ ምቹ ሁኔታዎች ምን ምን ናቸው? (ምርመራ፡- የተጨማሪ ምግብ አመጋገብ ባሀልን ክሚያጎለብቱ ልምዶች ጋር በተገናኘ የማህበረሰብ እና የጤና አጠባበቅ ስርዓት ድጋፍን እንዴት ያብራሩታል?

- እኔ ያሉኝን ጥያቄዎች ጨርሻለሁ። እስካሁን በተነጋገርነው ዙሪያ ቀረ የሚሉት ነገር ካለ እባክዎን በዝርዝር ይግለጹልኝ?

ስለ ትብብርዎ በጣም አመሰግናለሁ።

**English version:** Interview guides to assess barriers and enablers of complementary feeding practices among mothers for their children

**B) Key Informant (KI) Interview guide**

| **Participant’s background information** | | | | |
| --- | --- | --- | --- | --- |
| **Participant’s code** | **Age** | **Sex** | **Education** | **Marital status** |
| WDA (Women Development Army), HEW (Health extension worker) or HEO (Health Extension Officer) |  |  |  |  |

1. Knowledge about complementary feeding practices (CFP) (probe: What is child complementary feeding mean? (Probe: Why complementary foods (CF) are needed in addition to breast milk? What is the amount of CF a child feed at a time? Any influence regarding CF
2. What is appropriate complementary feeding mean? (Probe: When you can say complementary feeding is appropriate for a child? (in terms continuing breast milk, dietary diversity, meal frequency and egg &/or flesh food consumption).
3. How do you describe the challenges of ACF practices?

Probe:

- Preparing CF from various food sources (in terms of: cost, availability & time).
- How do you describe the culture of child feeding practices in this locality? (Probe: Any food taboo for this age group? Can you give example of food taboo during this age? Why this can be done?)
- How do you think about feeding animal source foods for a child below 2 years? (Probe: Can you give example of ASF to be given at this age? What are its benefits?)
- What do mothers think about preparing and feeding animal source foods during fasting periods?
- What are the persons’ thought (grandmother/father or community) about ACF practices?
- Why not mothers practice CF appropriately? (Probe: in terms of availability, cost, time, or working condition)

1. What are the things that make it possible for children to eat balanced diet? (Probe: How do you explain community and healthcare system support related to CF practices?)

- If anything, else regarding this issue, please describe it in detail?

Thank you for your time and cooperation!

**አማርኛ ቅጂ፡-** እናቶች ልጆቻቸውን የተመጣጠነ ተጨማሪ ምግብ ለመመገብ የማያስችሉ ተግዳሮቶችን እና የተመጣጠነ ምግብ ለመመገብ የሚያስችሉ ምቹ ሁኔታዎችን ለመገምገም የተዘጋጀ ምሪ ምጠይቅ።

**ለ) ኪ-ኢንፎርማንት ኢንተርቪው** (Key informant Interview guide)

| **በቃለ መጠይቁ የተሳተፉ ስዎች አጭር ምረጃ** | | | | |
| --- | --- | --- | --- | --- |
| **መለያ (ምልክት)** | **እድሜ** | **ዖታ** | **የት/ርት ደረጃ** | **የጋብቻ ሁኔታ** |
| የሴቶች የልማት ቡድን መሪ፣ የጤና ኤክስቴንሽን ባለሙያ፣ ወይም የጤና ኤክስቴንሽን አስተባባሪ |  |  |  |  |

1. ስለ ተጨማሪ መግብ አመጋገብ ልምድ እውቀት በተመለከተ (ምርመራ፡- የህጻን ተጨማሪ ምግብ ማለት ምን ማለት ነው? ከጡት ወተት በተጨማሪ ለህጻኑ ተጨማሪ ምግቦች ለምን ያስፈልጋሉ?) አንድ ህፃን በአንድ ጊዜ የሚመገበው የምግብ መጠን ምን ያክል ነው? በተጨማሪ ምግብ አመጋገብ በተመለከተ ምን ምን ተጽእኖ አለ?
2. የተመጣጣነ ተጨማሪ ምግብ ማለት ምን ማለት ነው? (ምርመራ፡- ገንፎ ክምን አይነት የእህል ስብጥር ሊዘጋጅ ይችላል? የተለያዩ የህፃናት የምግብ አይነቶች ስንል ምን ማለታችን ነው? ህጻኑ ተገቢ የሆነ ተጨማሪ ምግብ እየተመገበ ነው የሚሉት ምን ምን ምግቦችን ሲበላ ነው? (ምርመራ፡- የጡት ወተት ማጥባት መቀጠል፣ በእድሜው ልክ የተለያአዩ የምግብ አይነቶችን መመገብ፣ በቀን ወሰጥ ምግብ ደጋግሞ መመገብ፣ እና የእንስሳት ምንጭ ምግቦች (ወተት፣ እንቁላል፣ የሥጋ ምግብ)።
3. ከ 2 ዓመት በታች ያሉ ህፃናትን ተመጣጣኝ ተጨማሪ ምግቦችን ለመመገብ የማያስችሉ ተግዳሮቶችን እንዴት ይገልጿችዋል?

**ምርመራ፡-**

- ከተለያዩ የምግብ ምንጮች ተጨማሪ ምግብ ማዘጋጀት (ምርመራ፡- ከዋጋ አንጻር፣ ምግብ ለምስራት ከሚያገለግሉ አቅርቦቶች አንጻር (እህል፣ ጥራጥሬ፣ ወተት፣ ስጋ ፣ እንቁላል፣ በቫይታሚን-ኤ የበለፀጉ ፍራፍሬዎች እና አትክልቶች)።
- በአካባቢዎ ያለውን የተለመደ/ባህላዊ የህፃናት አመጋገብ ሁኔታ እንዴት ይገልጹታል? (ምርመራ፡- በዚህ እድሜ ውስጥ ላሉ ህጻናት የሚከለከሉ ምግቦች ይኖራሉ? የሚከለከሉ ምግቦች ካሉ ምሳሌ ሊጠቅሱልኝ ይችላሉ? እንዲህ ለምን ያደርጋሉ? ቢያአብራሩልኝ?)
- ከ **2** ዓመት በታች ላሉ ህፃናት የእንስሳት ምንጭ ምግቦችን በመመገብ ዙሪያ ምን ያስባሉ? (ምርመራ፡- በዚህ እድሜ ውስጥ ያሉ ህጻናት መብላት ያለባቸው የእንስሳት ምንጭ የሆኑ ምግቦች ምን ምን ናችው? ጥቅማቸውስ ምን ሊሆን ይችላል?)
- በጾም ወቅት የእንስሳት ምንጭ የሆኑ ምግቦችን ለህጻናት አዘጋጅቶ በመመገብ ዙሪያ ምን ይላሉ?
- ሰዎች ​​(ቤተሰብ፣ አያት/አባት፣ የአካባቢ ሰዎች) በሀጻናት የተመጣጠነ ተጨማሪ ምግብ አመጋገብ ዙሪያ ምን ዓይነት አመለካከት/አስተሳሰብ/ አላቸው?
- እናቶች የተመጣጥነ ተጨማሪ ምግብ አመጋገብ ስርዓትን በትክክል የማይከተሉት ለምን ነው? (ምርመራ፡- ምግብ ለምስራት ከሚያገለግሉ አቅርቦቶች አንጻር (እህል፣ ጥራጥሬ፣ ወተት፣ ስጋ፣ እንቁላል፣ በቫይታሚን-ኤ የበለፀጉ ፍራፍሬዎች እና አትክልቶች) እንዲሁም ከዋጋ፣ ጊዜ፣ የስራ ሁኔታ አንጻር ቢያብራሩልኝ) ።

1. ህጻናትን የተመጣጠነ ምግብ መመገብ የሚያስችሉ ምቹ ሁኔታዎች ምን ምን ናቸው? (ምርመራ፡- የተጨማሪ ምግብ አመጋገብ ባሀልን ክሚያጎለብቱ ልምዶች ጋር በተገናኘ የማህበረሰብ እና የጤና አጠባበቅ ስርዓት ድጋፍን እንዴት ያብራሩታል?

- እኔ ያሉኝን ጥያቄዎች ጨርሻለሁ። እስካሁን በተነጋገርነው ዙሪያ ቀረ የሚሉት ነገር ካለ እባክዎን በዝርዝር ይግለጹልኝ?

ስለ ትብብርዎ በጣም አመሰግናለሁ።
