# Supplementary material for: Appropriate complementary feeding practices and associated factors among mothers with infants aged 6–8 months in West Gojjam Zone, Northwest Ethiopia: A mixed methods study
Source: PLoS One. 2025 Oct 16;20(10):e0334740. doi: 10.1371/journal.pone.0334740 (PMC12530518; doi:10.1371/journal.pone.0334740)
Supplement: S2 Dataset — (PDF) [file pone.0334740.s003.pdf]

Synthesis 1: Knowledge

Line No	Text	Codes	Synthesis 1
	Document: Barriers of ACFP		
55	A participant stated: Oh… yes, the parent says prepare. In fact,	Influence on CF feeding	Knowledge
56	some parents even give resource. The parent also provides.	Influence on CF feeding	Knowledge
57	Then, we prepare and give it to the child.	Influence on CF feeding	Knowledge
146	This participant indicated: It's the resource, but the knowledge is	K. regarding CFP, Poor	Economy (Pove
147	there. Health workers also informing us when children should.	K. regarding CFP, Poor	Economy (Pove
148	start CFs	K. regarding CFP, Poor	Economy (Pove
203	Parents give what is available. Because it is for children. They give	ACFP, Influence on CF feeding	Dietary diversity
204	what is found. There is no elder family influence. There is no	ACFP, Influence on CF feeding	Dietary diversity
205	influence.	ACFP, Influence on CF feeding	Dietary diversity
331	A second participant shared: Yes... The parent is going to a work	Influence on CF feeding	Knowledge
332	setting, we have no resource. So, we are giving that we have and	Influence on CF feeding	Knowledge
333	sit. Where does the income from? If work started, of course, we	Influence on CF feeding	Knowledge
334	would work and grow up, and our child would not be abused.	Influence on CF feeding	Knowledge
335	Now, the parent is going; the child will eat what I eat. That is, it.	Influence on CF feeding	Knowledge
506	A third participant described: It is porridge and soup-like liquid	ACFP, K. regarding CFP	Dietary diversity
507	made from grains.	ACFP, K. regarding CFP	Dietary diversity
520	This participant further explained: It is, as far as the child satisfied.	Amount of CF at a time, K. regarding CFP	Dietary diversity
521	The precise amount is unknown, but feed until the child satisfied	Amount of CF at a time, K. regarding CFP	Dietary diversity
522	(refuses). At six months, it was given about 3 to 4 with small size	Amount of CF at a time, K. regarding CFP	Dietary diversity
523	spoonsful.	Amount of CF at a time, K. regarding CFP	Dietary diversity
602	This participant also said: It could be unlearning.	K. regarding CFP	Knowledge
798	Another participant stated: It is not because of seeing someone	Influence on CF feeding	Knowledge
799	who gives CF, but because of the progress of time, the current life	Influence on CF feeding	Knowledge
800	is different from the past. So, since health workers informed us to	Breastfeeding, Influence on CF feeding	Continue breast
801	start CF after six months, we will also breastfeed the child as much	Breastfeeding, Influence on CF feeding	Continue breast
802	as we can until six months, and after that, we prepare porridge flour	Influence on CF feeding, Time to start CF	Knowledge, Ti
803	ahead of time and we try to feed one spoon and continue like this.	Influence on CF feeding, Time to start CF	Knowledge, Ti
804	The improvement, the state of time, awakening, the present person	Influence on CF feeding, Time to start CF	Knowledge, Ti
805	and the old person are not equal. It is getting better.	K. regarding CFP	Knowledge
806	It is about the situation of time; it is not seeing a person who	K. regarding CFP	Knowledge
807	does the action or not.	K. regarding CFP	Knowledge
823	This participant noted: As mentioned earlier, there is nothing other	K. regarding CFP	Knowledge
824	than what it was described.	K. regarding CFP	Knowledge

Line No	Text	Codes	Synthesis 1
871	such a thing from anyone in the family or community we do the best	Influence on CF feeding, People's opinion on ACFP	Attitude, Knowl
872	for the child, no one has influencing in CF. No one can control	Influence on CF feeding	Knowledge
873	except the lack of time.	Influence on CF feeding	Knowledge
903	They also said: This is one of the several children. Others grew not	K. regarding CFP	Knowledge
904	like this child. This is a more modern approach. That is modified.	K. regarding CFP	Knowledge
905	Laugh… The elder children grow up eating what the family ate.	K. regarding CFP	Knowledge
1061	A fifth individual shared: Yes, there is an influencer. It is because	Influence on CF feeding	Knowledge
1062	we are faced with it. For example, individuals say, we grew up	Influence on CF feeding	Knowledge
1063	eating fermented flatbread with legume-based dish, where is this child	Influence on CF feeding	Knowledge
1064	from? Whether the child eats or not, can grow up to what the God	Influence on CF feeding	Knowledge
1065	says. It is said that "A child grows by opportunity". A child grows by	Influence on CF feeding	Knowledge
1066	opportunity means, the child eats what is available, what the	Influence on CF feeding	Knowledge
1067	opportunity gives, and grows up to what was created to be.	Influence on CF feeding	Knowledge
1068	This idea is from male parents.	Influence on CF feeding	Knowledge
1072	This individual added: Because male parents don't have enough	K. regarding CFP	Knowledge
1073	knowledge. Don't have enough knowledge means, when a mother	K. regarding CFP	Knowledge
1074	feed the child at the age of 6 months, at that time they say you	K. regarding CFP	Knowledge
1075	choke the child, will be choked. It's just that there are parents who	K. regarding CFP	Knowledge
1076	reverses the lessons learnt. There are who say that: in 6	K. regarding CFP	Knowledge
1077	months...? the child should be one year, what is going to eat	K. regarding CFP	Knowledge
1079	now? the child does not have teeth, and cannot swallow.	K. regarding CFP	Knowledge
1096	Participant 5: It is a body building and repairing, disease-fighting	K. regarding CFP	Knowledge
1097	and energy and heat-giving content foods.	K. regarding CFP	Knowledge
1139	This participant noted: If not lack of knowledge, no challenge. It is	K. regarding CFP	Knowledge
1140	about lack of knowledge and acceptance. Parents say there is	K. regarding CFP	Knowledge
1141	nothing to be grow or sell in the area. But there is a lack of	K. regarding CFP	Knowledge
1241	knowledge.	K. regarding CFP	Knowledge
1143	That is, when you ask a parent to provide CF or cut and give a	Lack of K. of fru./veget. Foods	Knowledge
1144	banana, they say rather than giving banana, it is better if we sell &	Lack of K. of fru./veget. Foods	Knowledge
1145	buy some onion or oil. Not knowing the benefits of bananas,	Lack of K. of fru./veget. Foods	Knowledge
1146	vegetables, fruits. For example, if there is cabbage, they will sell it,	Lack of K. of fru./veget. Foods	Knowledge
1147	and bring potato or use it for other expenses	Lack of K. of fru./veget. Foods	Knowledge
1149	There is a lack of knowledge, a lack of understanding.	Lack of K. of fru./veget. Foods	Knowledge
1150	There is a problem of attitude to CF practice. It has nothing else.	Lack of K. of fru./veget. Foods	Knowledge

Line No	Text	Codes	Synthesis 1
1254	This participant also highlighted: Although the decision maker for	Influence on CF feeding, People’s opinion on ACFP	Attitude, Knowl
1255	CF is the primary	Influence on CF feeding, People’s opinion on ACFP	Attitude, Knowl
1256	caregiver, parents also contributed.	Influence on CF feeding, People’s opinion on ACFP	Attitude, Knowl
1257	There are people who say, the child will grow up if eats this.	Influence on CF feeding, People’s opinion on ACFP	Attitude, Knowl
1258	The then is another day and now is another day. The then and	Influence on CF feeding, People’s opinion on ACFP	Attitude, Knowl
1259	children of today are different. These persons say, "Give this,	Influence on CF feeding, People’s opinion on ACFP	Attitude, Knowl
1260	feed this food."	Influence on CF feeding, People’s opinion on ACFP	Attitude, Knowl
1261	The individuals inform the mother, you should understand what	Influence on CF feeding, People’s opinion on ACFP	Attitude, Knowl
1264	counselors tell you. In the past, it was different and now it is	Influence on CF feeding, People’s opinion on ACFP	Attitude, Knowl
1265	different and accept what science has brought. There are also	Influence on CF feeding, People’s opinion on ACFP	Attitude, Knowl
1266	negative ones. Especially there are problems with male parents.	Influence on CF feeding, People’s opinion on ACFP	Attitude, Knowl
1267	It may be because of counselling pay attention to mothers.	Influence on CF feeding, People’s opinion on ACFP	Attitude, Knowl
1268	Males say we are farmers; we dig maize, the child should	Influence on CF feeding, People’s opinion on ACFP	Attitude, Knowl
1269	eat what is available, “Are we going to go buy vegetables?” We are	Influence on CF feeding, People’s opinion on ACFP	Attitude, Knowl
1270	digging there, and “Is the mother sit down with a baby?”	Influence on CF feeding, People’s opinion on ACFP	Attitude, Knowl
1288	This participant also explained: Because there is a lack of	K. regarding CFP, Lack of time	Knowledge, Wo
1289	knowledge. Secondly, Dung gives birth means; male parents	K. regarding CFP, Lack of time	Knowledge, Wo
1317	do not have any knowledge of a child. It just ended.	Gender role, K. regarding CFP, Men’s attitude to CFP	Attitude, Knowl
1318	Baby is born, they say thank you and accept the baby, but nothing	Gender role, K. regarding CFP, Men’s attitude to CFP	Attitude, Knowl
1319	about “What does a baby want?” Let alone six months, the male	Gender role, K. regarding CFP, Men’s attitude to CFP	Attitude, Knowl
1320	parents said, "What is the benefit of vaccination from 45 days?"	Gender role, K. regarding CFP, Men’s attitude to CFP	Attitude, Knowl
1400	Because parents are outdoor workers. Since the parents’ focus	Influence on CF feeding	Knowledge
1402	is on work, this can be an obstacle. But it's not always an obstacle.	Influence on CF feeding	Knowledge
1403	Family, especially when the parent goes to work setting, the child	Influence on CF feeding	Knowledge
1404	is given to the elder child, and when the	Influence on CF feeding	Knowledge
1405	elder child goes to work setting, mother stays at home,	Influence on CF feeding	Knowledge
1406	So, there is not much hindrance. There may be a fifty percent	Influence on CF feeding	Knowledge
1407	barrier. But parents don't give CFs to children completely (it's not	Influence on CF feeding	Knowledge
1408	balanced), they give what the household produced.	Influence on CF feeding	Knowledge
1475	In terms of preparation, there are many parents who are	CF preparation, Lack of education	Food preparatio
1476	uneducated, illiterate. They said that those who give it like a	CF preparation, Lack of education	Food preparatio
1477	fermented flatbread? who give what the house produced? who do	CF preparation, Cleanliness, Lack of education	Food preparatio
1478	keep their cleanliness? There is something that happens without	CF preparation, Cleanliness, Lack of education	Food preparatio
1479	learning. There is no food problem as a farmer now.	CF preparation, Foodstuffs related, Lack of education	Agriculture prod

Line No	Text	Codes	Synthesis 1
1550	A participant observed: From the good advice, people advise parents to	Influence on CF feeding, People’s opinion on ACFP	Attitude, Knowl
1551	feed, drink and clean children on time. On the other hand, people also	Influence on CF feeding, People’s opinion on ACFP	Attitude, Knowl
1552	say no... why do you do this? Why are you doing this? They	Influence on CF feeding, People’s opinion on ACFP	Attitude, Knowl
1553	say, “This is nonsense. We know this thing. Sop this talk.”	Influence on CF feeding, People’s opinion on ACFP	Attitude, Knowl
1554	The person who says that “we know it” is said to be	Influence on CF feeding, People’s opinion on ACFP	Attitude, Knowl
1555	contemptuous and mocking. Especially when we go to participants’	Influence on CF feeding, People’s opinion on ACFP	Attitude, Knowl
1556	homes, there are some individuals express strong defiance	Influence on CF feeding, People’s opinion on ACFP	Attitude, Knowl
1557	or superiority. Because there are many. One is bad, one is rough, most	Influence on CF feeding, People’s opinion on ACFP	Attitude, Knowl
1558	have no have good attitude.	Influence on CF feeding, People’s opinion on ACFP	Attitude, Knowl
1559	When one says right thing, one twists it. When one speaks	Influence on CF feeding, People’s opinion on ACFP	Attitude, Knowl
1560	the right, the other brings the left. They say because one of their	Influence on CF feeding, People’s opinion on ACFP	Attitude, Knowl
1561	views are bad. Two is a matter of unlearning. They say that because	Influence on CF feeding, People’s opinion on ACFP	Attitude, Knowl
1562	either their view is bad or it is a matter of unlearning. The second	Lack of education, People’s opinion on ACFP	Attitude, Knowl
1564	is to just go the random, fill-the-dash. It is just going to be useless.	Lack of education, People’s opinion on ACFP	Attitude, Knowl
1565	It means without knowledge. For example, what does it matter? the	Lack of education, People’s opinion on ACFP	Attitude, Knowl
1566	child will grow up regardless, or the child will find own way.	Lack of education, People’s opinion on ACFP	Attitude, Knowl
1639	Because even if family do not bring food items from the market, even if	K. regarding CFP, Type of CF the child feeds	Dietary diversity
1640	they have a knowledge gap; it is the community that advise eat what is	K. regarding CFP, Type of CF the child feeds	Dietary diversity
1709	available. Lack of knowledge. Some individuals say the child cannot eat	K. regarding CFP	Knowledge
1710	CFs, and those who give their breasts only advise “the child’s capacity	K. regarding CFP	Knowledge
1711	does not suit for CFs.” They say that the child can eat only after	K. regarding CFP	Knowledge
1712	one year. Others say that the stool will increase.	CF prohibition related, K. regarding CFP	Knowledge, Mis
1733	One participant described: First from the breast, then after porridge	K. regarding CFP	Knowledge
1734	made from a local grain and other things added. If the child eats any	K. regarding CFP	Knowledge
1735	food, the child will become sick.	K. regarding CFP	Knowledge
1865	This participant added: Parents knowledge is developing; they say	K. regarding CFP	Knowledge
1866	children were abused and children have not been breastfed and the	K. regarding CFP	Knowledge
1867	parents are suffering with work. Parents prepare; it is mandatory.	K. regarding CFP	Knowledge
1978	A participant indicated: It is a primary caregiver. There is no other	Decision maker of CF, Influence on CF feeding	Knowledge, Ti
1979	family who influences negatively because this is the child. There will	Decision maker of CF, Influence on CF feeding	Knowledge, Ti
1980	not be.	Decision maker of CF, Influence on CF feeding	Knowledge, Ti
1987	This participant added: Oh… can be a weakness of thinking. And	K. regarding CFP	Knowledge
1988	secondly, it is the child going to eat cereal from now on...? There are	K. regarding CFP	Knowledge
1989	parents who say like this.	K. regarding CFP	Knowledge

Line No	Text	Codes	Synthesis 1
2061	An individual observed: They have knowledge. They are just	Info. access of information to IYCFP, K. regarding CFP	Knowledge, Net
2062	getting started. They are giving and their knowledge is good. There	Info. access of information to IYCFP, K. regarding CFP	Knowledge, Net
2062	are vegetables,	Info. access of information to IYCFP, K. regarding CFP	Knowledge, Net
2200	fruits, whatever, if it is not a problem of knowledge and time, they	Foodstuffs related, K. regarding CFP	Agriculture prod
2201	are willing. One will not find.	Foodstuffs related, K. regarding CFP	Agriculture prod
2300	An individual stated: Nowadays, they are gaining knowledge	K. regarding CFP	Knowledge
2301	through education. Though parents understand what it should be,	K. regarding CFP, Negligence	Attitude, Knowl
2302	there are also parents that you will find before they start CFs.	K. regarding CFP, Negligence	Attitude, Knowl
2303	When you ask parents what is the reason, you will find	K. regarding CFP, Negligence	Attitude, Knowl
2309	But many parents know when CF be started and the benefits	K. regarding CFP	Knowledge
2310	of complementary foods.	K. regarding CFP	Knowledge
2371	A participant recounted: When health workers asked parents who	Influence on CF feeding	Knowledge
2372	started CF early, before they received education, or who were	Influence on CF feeding	Knowledge
2373	pushed by elder family individuals and started before 6 months,	Influence on CF feeding	Knowledge
2374	respond that “this is an advice by elder family member.” No..., for	Influence on CF feeding	Knowledge
2375	example, the child was given a specific early food just after birth,	Influence on CF feeding	Knowledge
2376	the child grows with milk like this.	Influence on CF feeding	Knowledge
2377	There are many mothers who start cow milk especially	Influence on CF feeding	Knowledge
2378	before 6 months. And there is. There are elder families who	Influence on CF feeding	Knowledge
2379	say like that. But then again, parents advised as it not right.	Influence on CF feeding	Knowledge
2380	What is started, should be stopped. Health workers teach parents	Influence on CF feeding	Knowledge
2381	that CFs should not be started before 6 months during the next	Influence on CF feeding	Knowledge
2382	birth. But next time (when parent gives birth again), that parent will	Influence on CF feeding	Knowledge
2383	not start food before 6 months. not, the parent will decide. Male	Influence on CF feeding	Knowledge
2534	parents have no experience of negative effect regarding CF.	Decision maker of CF, Influence on CF feeding	Knowledge, Ti
2535	But it cannot be said that the male parent will not willing	Influence on CF feeding	Knowledge
2536	to be the child’s growth is age appropriate. There may be	Influence on CF feeding	Knowledge
2537	absence of a supply. Otherwise, parents did not experience	Influence on CF feeding	Knowledge
2663	any influence by another person on the child's complementary	Influence on CF feeding, People’s opinion on ACFP, Poor	Attitude, Econo
2664	intake.	Influence on CF feeding	Knowledge
2875	An individual stated: One thing that keeps parents from giving CF	Lack of education	Knowledge
2876	is often uneducated. Another shortage of time. It is a problem of	Lack of education, Lack of time	Knowledge, Wo
2886	knowledge.	K. regarding CFP, Lack of attention to CFP	Attitude, Knowl
2904	This individual shared: There are parents who know very well how	K. regarding CFP	Knowledge
2905	to feed complementary foods. Apart to this, there are some parents	K. regarding CFP, Reluctance	Attitude, Knowl
2906	who do not accept even after they have been informed	Lack of education, Reluctance	Attitude, Knowl

Line No	Text	Codes	Synthesis 1
2907	Emphasis is not given due to lack of knowledge, because they	Lack of education, Reluctance	Attitude, Knowl
2908	do not pay attention to CF. If parents are educated, they are likely	Lack of education, Reluctance	Attitude, Knowl
2909	to accept the guidance sooner. They acknowledge it is for the	Lack of education	Knowledge
2910	Child. Those uneducated parents will not understand quickly when	Lack of education	Knowledge
2911	informed, it may take time. Of course, they may accept after a	Lack of education	Knowledge
2912	while. A small percentage of caregivers may not accept the	K. regarding CFP	Knowledge
2913	information provided. A parent may say no time, not to act too soon	K. regarding CFP	Knowledge
2914	even if they are informed, but when informed about the benefits for	K. regarding CFP	Knowledge
2915	their child prevents from illness, the child will be good at,	K. regarding CFP	Knowledge
2917	education, thinking the information will be accepted. But it may	K. regarding CFP	Knowledge
2918	take some time for unresponsive parents to take the information	K. regarding CFP	Knowledge
2919	and implement. Apart from this, responsive parents also say, of	K. regarding CFP	Knowledge
2921	please inform us your visit, come and look, we will prepare CF to	K. regarding CFP	Knowledge
2922	the child for your observation.	K. regarding CFP	Knowledge
3167	This participant added: Same, cognitive (knowledge) problem.	K. regarding CFP, Meal Frequency	Frequency of C
3168	After preparing the porridge flour, the problem is giving it in time.	K. regarding CFP, Meal Frequency	Frequency of C
3226	They also noted: Because parents see their tasks. Many times,	Attitude to CFP, K. on the consequences of u.n	Attitude, Knowl
3227	since children spend time with primary caregivers, they do not	Attitude to CFP, K. on the consequences of u.n	Attitude, Knowl
3228	aware the child’s food shortage. So, there is a problem of not	Attitude to CFP, K. on the consequences of u.n	Attitude, Knowl
3229	understanding the food shortage it will cause in the future. If,	Attitude to CFP, K. on the consequences of u.n	Attitude, Knowl
3230	children breastfeed & if parents give something, that it is enough.	Attitude to CFP, K. on the consequences of u.n	Attitude, Knowl
3335	A participant further explained: Then, it is a problem of	K. on the consequences of u.n, Negligence	Attitude, Knowl
3336	implementation. It is still an understanding problem. It is perceived	K. on the consequences of u.n	Knowledge
3337	as a problem of not understand well how much the actual problem	K. on the consequences of u.n	Knowledge
3338	[undernutrition] can be caused due to lack of food. Because it may	K. on the consequences of u.n	Knowledge
3339	be thought undernutrition is not going to happen, and there is also,	K. on the consequences of u.n	Knowledge
3340	the attitude that the previous peoples have grown up like this.	K. on the consequences of u.n, Negligence	Attitude, Knowl
3341	When pressure is applied to prepare CFs, parents say "The child	K. on the consequences of u.n, Negligence	Attitude, Knowl
3342	is” unwilling to eat It is searching for a reason. The child is not	K. on the consequences of u.n, Negligence	Attitude, Knowl
3343	willing to feed.	K. on the consequences of u.n, Negligence	Attitude, Knowl
3451	A participant stated: As it is known in this context, the male	Influence on CF feeding	Knowledge
3452	parents usually spend the time mostly outside, so they do not have	Influence on CF feeding	Knowledge
3453	pressure on the child because the primary caregiver is close	Influence on CF feeding	Knowledge
3454	to the child. In this context, unless there is a difference in skills	Influence on CF feeding	Knowledge
3455	between parents, male parents have no such an impact.	Influence on CF feeding	Knowledge
3459	This participant added: It is thought that parents do not start	K. regarding CFP	Knowledge

Line No	Text	Codes	Synthesis 1
3460	additional [complementary] feeding at 6m mainly because of the	K. regarding CFP	Knowledge
3461	knowledge gaps. First, because parents believe that children	K. regarding CFP	Knowledge
3462	might not take the food that is prepared. Second, thinking that	BM is enough, K. regarding CFP	Knowledge, Mis
3463	breast milk is enough for the child. Third, because of not	BM is enough, Lack of K. of nutritious foods	Knowledge, Mis
3464	understand the health benefits that the child can get from	Lack of K. of nutritious foods	Knowledge
3465	complementary foods. Due to these 3 reasons, there is a	Lack of K. of nutritious foods	Knowledge
3466	tendency not to start CFs on time.	Lack of K. of nutritious foods	Knowledge
3513	A participant further detailed: As it is explained, the challenges	K. regarding CFP	Knowledge
3514	are often the same. Reasons why children under 2 years of age	K. regarding CFP	Knowledge
3515	do not receive appropriate (adequate) complementary foods:	K. regarding CFP	Knowledge
3516	First, parents' knowledge. Parents' or guardians' knowledge of	K. regarding CFP	Knowledge
3517	what items an appropriate complementary food can be a	K. regarding CFP	Knowledge
3518	challenge. A well-informed parent can give the complementary	K. regarding CFP	Knowledge
3519	food well. Those who do not have good knowledge cannot give	K. regarding CFP	Knowledge
3520	complementary food. It will be a challenge especially if parents	K. regarding CFP	Knowledge
3521	do not have the knowledge of the frequency, composition, portion	K. regarding CFP	Knowledge
3522	size, thickness, and the procedure (hygiene). Second, it	K. regarding CFP	Knowledge
3523	could be the economy.	K. regarding CFP, Poor	Economy (Pove
3559	But most of the time, parents are still not fully implemented	Info. access of information to IYCFP, Lack of K. of nutritious foods	Knowledge, Net
3560	this behavioral change. It is called what the household produces. So	Lack of K. of nutritious foods	Knowledge
3561	what does it often do, by taking out the things that are in the house	Attitude to change foodstuffs, Lack of K. of nutritious foods	Attitude, Knowl
3562	and buying other things. But the practice of preparing and giving	Attitude to change foodstuffs, Lack of K. of nutritious foods	Attitude, Knowl
3563	these food groups to children are weak. For example, if there are	Attitude to change foodstuffs, Lack of K. of nutritious foods	Attitude, Knowl
3564	things like eggs that are good for children, they take to the	Attitude to change foodstuffs, Lack of K. of nutritious foods	Attitude, Knowl
3565	market for sell, and buy other simple things (less nutritious items).	Attitude to change foodstuffs, Lack of K. of nutritious foods	Attitude, Knowl
3566	For example, if you have eggs or honey at home, there is	Attitude to change foodstuffs, Lack of K. of nutritious foods	Attitude, Knowl
3567	something to sell and buy other simple things (less nutritious).	Attitude to change foodstuffs, Lack of K. of nutritious foods	Attitude, Knowl
3568	They don't understand the food ingredients between	Attitude to change foodstuffs, Lack of K. of nutritious foods	Attitude, Knowl
3569	those sold and those bought. It is compared to the profits made.	Attitude to change foodstuffs, Lack of K. of nutritious foods	Attitude, Knowl
3570	And the practice of eating proper or nutritious food is not yet	Attitude to change foodstuffs, Lack of K. of nutritious foods	Attitude, Knowl
3571	developed and still have a lot to be done.	Attitude to change foodstuffs, Lack of K. of nutritious foods	Attitude, Knowl
3578	This participant commented: This context still needs a lot of work.	Attitude to change foodstuffs, Lack of K. of nutritious foods	Attitude, Knowl
3579	There are gaps regarding to feeding these animal products to	Attitude to change foodstuffs, Lack of K. of nutritious foods	Attitude, Knowl
3580	children. Because now if there is an egg, it is to sell the egg and	Attitude to change foodstuffs, Lack of K. of nutritious foods	Attitude, Knowl
3581	convert it into money. If there is milk, it is to turn the milk into	Attitude to change foodstuffs, Lack of K. of nutritious foods	Attitude, Knowl
3582	butter which is more expensive than milk, so instead of eating butter	Attitude to change foodstuffs, Lack of K. of nutritious foods	Attitude, Knowl
3583	at home, it is sold and the money used for other purposes.	Attitude to change foodstuffs, Lack of K. of nutritious foods	Attitude, Knowl
3584	This must be effectively implemented (improved).	Attitude to change foodstuffs, Lack of K. of nutritious foods	Attitude, Knowl

Line No	Text	Codes	Synthesis 1
3634	A participant mentioned: As previously stated, it is impossible to	K. regarding CFP	Knowledge
3635	say that the knowledge of parents or caregivers is complete by	K. regarding CFP	Knowledge
3636	preparing and giving these extra meals. From their perspective,	K. & practice mismatch, K. regarding CFP	Attitude, Knowl
3637	there are gaps in the context practice and knowledge. It is related to	K. & practice mismatch, K. regarding CFP	Attitude, Knowl
3638	perception and knowledge and problems in implementation.	K. & practice mismatch, K. regarding CFP	Attitude, Knowl
3801	A participant affirmed: Yes, it is. For example, it could be an elder	Influence on CF feeding	Knowledge
3802	family Member or a friend. Uh... well sometimes like that. There can	Influence on CF feeding	Knowledge
3803	be a similar negative effect on the entire family.	Influence on CF feeding	Knowledge
3806	This participant stated: That could be a lack of awareness. There is	Influence on CF feeding	Knowledge
3807	a fundamental problem that the community is facing in terms of	Influence on CF feeding	Knowledge
3808	child feeding. In fact, it should be taken in to account about and	Influence on CF feeding	Knowledge
3809	what is required to be, but when it is deeply seen, parents may	Influence on CF feeding	Knowledge
3810	not respect what they have told to start CFs at six months.	Influence on CF feeding	Knowledge
3811	There are parents who give complementary foods after birth, and	Influence on CF feeding, Negligence	Attitude, Knowl
3812	there are parents who give it at four months. That is very	Influence on CF feeding, Negligence	Attitude, Knowl
3814	difficult, and this practice is persisted still.	Influence on CF feeding, Negligence	Attitude, Knowl
3817	An individual expressed: It is just a problem of attitude. Lack of	Influence on CF feeding, Lack of education	Knowledge
3818	awareness, lack of education (not being educated) is a big problem	Influence on CF feeding, Lack of education	Knowledge
3819	in the community. Now in fact, what seems practical is to start	Influence on CF feeding, Lack of education	Knowledge
3820	early. The discussion concerns about the main and what the science	Influence on CF feeding, Lack of education	Knowledge
3821	says, but what is being practically applied to the community is to	Influence on CF feeding, Lack of education	Knowledge
3822	start early [before 6 months]. It is common, it is given.	Influence on CF feeding, Lack of education	Knowledge
3823	Firstly, there is a lack of awareness in the community.	Influence on CF feeding, Lack of education	Knowledge
3824	Uh... there is traditional practice. There are also negative effects by	Influence on CF feeding, Lack of education	Knowledge
3825	another individual. The belief is that if complementary feeding is not	Influence on CF feeding	Knowledge
3826	introduced early, the child may face feeding difficulties later.	Influence on CF feeding	Knowledge
3827	Because of this, it is believed that children should become	Influence on CF feeding	Knowledge
3828	accustomed to it [CF] early. Because of the influences of parents	Influence on CF feeding	Knowledge
3829	or guardian insisting to start CF before 6 months.	Influence on CF feeding	Knowledge
3836	A participant stated: As previously mentioned, after 6 months, this	K. regarding CFP	Knowledge
3837	may not be the case for all communities. Sometimes parents may	K. regarding CFP	Knowledge
3838	not even give CF after 6 months. Parents said, CFs could not be	K. regarding CFP	Knowledge
3839	provided at six months, eight months, nine months, ten months.	K. regarding CFP	Knowledge
3840	As it was mentioned, there is a lack of knowledge and lack of	K. regarding CFP	Knowledge
3841	understanding from the local community practices, and customs.	K. regarding CFP	Knowledge
3842	It is not only starting CF early, but also starting lately. Lack of	K. regarding CFP	Knowledge

Line No	Text	Codes	Synthesis 1
3843	community awareness, saying, children's feces smell and	K. regarding CFP	Knowledge
3844	will increase. This is a serious lack of community awareness.	K. regarding CFP	Knowledge, Mis
3963	This participant explained: The challenges (barriers)... As	K. regarding CFP	Knowledge
3964	previously mentioned, the first is the lack of awareness.	Can't implement ACFP, K. regarding CFP	Knowledge, Mis
3965	Lack of awareness can be expressed in different ways. For	Can't implement ACFP, K. regarding CFP	Knowledge, Mis
3966	example, if health workers do not inform parents, they may think	Can't implement ACFP, K. regarding CFP	Knowledge, Mis
3967	about vegetables, grains, fruits, cereals, milk, and milk	Can't implement ACFP, K. regarding CFP	Knowledge, Mis
3968	products, and another animal source foods. Then, it is believed	Can't implement ACFP, K. regarding CFP	Knowledge, Mis
3969	that it will not be obtained and leave items available at home	Can't implement ACFP, K. regarding CFP	Knowledge, Mis
3970	and look elsewhere. It is also expected to explain well so that	Can't implement ACFP, K. regarding CFP	Knowledge, Mis
3971	parents don't have the view that can't be affordable.	Can't implement ACFP, K. regarding CFP	Knowledge, Mis
3972	There is a lot to say. Caregivers may perceive that if certain items	Can't implement ACFP, K. regarding CFP	Knowledge, Mis
3973	are unavailable, they are unable to afford purchasing others from the	Can't implement ACFP, K. regarding CFP	Knowledge, Mis
3974	market. They may perceive, if this is not available, without this,	Can't implement ACFP, K. regarding CFP	Knowledge, Mis
3975	we cannot afford to buy these from market. Consequently,	Can't implement ACFP, K. regarding CFP	Knowledge, Mis
3976	feelings of despair (hopelessness) regarding the ability to	Can't implement ACFP, K. regarding CFP	Knowledge, Mis
3977	perform the task may arise. The main challenge is lack of	Can't implement ACFP, K. regarding CFP	Knowledge, Mis
3978	awareness. The problem of not taking care of children.	Can't implement ACFP, K. regarding CFP	Knowledge, Mis
3985	A participant remarked: In terms of price, perhaps parents often	Attitude to change foodstuffs, Lack of K. of nutritious foods	Attitude, Knowl
3986	prefer to sell the available items, such as eggs and milk from	Attitude to change foodstuffs, Lack of K. of nutritious foods	Attitude, Knowl
3987	home. Instead of eating it, often it is a matter of taking to the	Attitude to change foodstuffs, Lack of K. of nutritious foods	Attitude, Knowl
3988	market and selling. Meaning, instead of giving these things to	Attitude to change foodstuffs, Lack of K. of nutritious foods	Attitude, Knowl
3989	children, thinking these things to the market, sell and earn money	Attitude to change foodstuffs, Lack of K. of nutritious foods	Attitude, Knowl
3990	and fulfill other programs, which is one of the challenges.	Attitude to change foodstuffs, Lack of K. of nutritious foods	Attitude, Knowl
3991	That is, because parents think that instead of giving these things	Attitude to change foodstuffs, Lack of K. of nutritious foods	Attitude, Knowl
3992	to children, selling and earn money and fulfill other	Attitude to change foodstuffs, Lack of K. of nutritious foods	Attitude, Knowl
3993	programs. This is one of the challenges. In terms of knowledge,	Attitude to change foodstuffs, Lack of K. of nutritious foods	Attitude, Knowl
3994	when the community is asked, there is a contented (aware)	Attitude to change foodstuffs, Lack of K. of nutritious foods	Attitude, Knowl
4032	person that explains or talks more about how children should	Attitude to CFP, K. regarding CFP	Attitude, Knowl
4033	be feed. When mothers' actual feeding practice is seen, there is a	K. regarding CFP	Knowledge
4034	problem in this regard. Because it is a lack of awareness.	K. & practice mismatch, K. regarding CFP	Attitude, Knowl
4100	This participant noted: The community have no that much	K. regarding CFP	Knowledge
4101	awareness (deep knowledge) about appropriate complementary	K. regarding CFP	Knowledge
4102	feeding as different food groups required: cereals, grains, fruits	K. regarding CFP	Knowledge
4103	vegetables, and animal source foods.	K. regarding CFP	Knowledge

Line No	Text	Codes	Synthesis 1
4104	Everything is from grains, legumes, vegetables, fruits, animal	K. regarding CFP	Knowledge
4105	products; Especially, about the 3 food ingredients; There is no	K. regarding CFP	Knowledge
4106	such deep thinking in the community regarding getting the	K. regarding CFP	Knowledge
4107	things that are called disease-preventing, nutritious,	K. regarding CFP	Knowledge
4108	energy and warmth. But there is not much problem in feeding	K. regarding CFP	Knowledge
4109	the food obtained.	K. regarding CFP	Knowledge
4263	Participant 15: Perhaps there are influences, especially in	Influence on CF feeding	Knowledge
4264	the areas where parents who do not receive counseling	Influence on CF feeding	Knowledge
4265	services, such problems arise.	Influence on CF feeding, Info. access of information to IYCFP	Knowledge, Net
4363	The reason is a cognitive (understanding) problem.	CF prohibition related, K. regarding CFP	Knowledge, Mis
4364	There is a situation where parents with family understanding	K. regarding CFP	Knowledge
4365	problems in CF do not start.	K. regarding CFP	Knowledge
4366	But there is no situation where this	CF prohibition related, K. regarding CFP	Knowledge, Mis

Synthesis 1: Dietary diversity

Line No	Text	Codes	Synthesis
	Document: Barriers of ACFP		
25	Participant 1: That is enough from vegetables, grains, Uh... that	Ingredients of porridge	Di
26	is from all types.	Ingredients of porridge	Di
47	Like local grain, barley, wheat, from different types, all kinds	Ingredients of porridge	Di
48	like beans, rice, and corn. We add all. We add four types like that.	Ingredients of porridge	Di
79	First with local grain, maize (corn), eh…the curd bean, uh... what	Ingredients of porridge	Di
80	rice also added. That is how CF started.	Ingredients of porridge	Di
93	Participant 1: We do not know much about diverse diets. It is	ACFP	Di
94	when the child breastfeeds and takes the porridge.	ACFP	Di
97	Participant 1: Because we are farmers. We do not know about	ACFP	Di
98	urban areas. But when the child eats porridge and breastfeeds,	ACFP	Di
99	we consider it appropriate food.	ACFP	Di
103	Participant 1: Now the child eats fermented flatbread with legume-	Type of CF the child feeds	Di
104	based dish. If it is available, also tastes bread, but does not eat	Meal Frequency, Type of CF the child feeds	Di
114	much foods. Participant 1: The amount is not known. It may be	Amount of CF at a time	Di
115	three small boluses. If the child eats	Amount of CF at a time, Meal Frequency	Di
124	Participant 1: No. There is nothing. There is no such thing. We	ASF feeding practices, Fru. & veg. feeding P.	Di
125	just give for the child what we can find, because we do not have	ASF feeding practices, Fru. & veg. feeding P.	Di
126	any. We do not have much.	ASF feeding practices, Fru. & veg. feeding P.	Di
129	Participant 1: We do not know that [diverse diets], only urban	Variety of foods	Di
130	people know. We are just offering only fermented flatbread with	Variety of foods	Di
131	legume-based dish. But we do not give anything like fruit.	Variety of foods	Di
135	Participant 1: Since the household does not have eggs, it is still	ASF feeding practices	Di
136	thinking about it, but the child did not consume diverse diet until now,	ASF feeding practices	Di
137	such as fruits (bananas, mangoes etc.). Eating like	Fru. & veg. feeding P.	Di
138	this is unusual. We don't know if whether the child needs meat and	Fru. & veg. feeding P.	Di
139	meat products. It will be in the future but now nothing.	ASF feeding practices	Di
140	On annual holidays, such as Christmas, we bring small meat.	ASF feeding practices	Di
141	When we eat, we give the fried meat for the child, but at other	ASF feeding practices	Di
142	times, meat is not available.	ASF feeding practices	Di

Line No	Text	Codes	Synthesis1
158	Participant 1: Sometimes we cooked legume flour in the form of	Type of CF the child feeds	Di
159	local legume-based dish, sometimes legumes [raw legumes] will be	Type of CF the child feeds	Di
160	boiled and given to children after the surface [cover] is removed.	Type of CF the child feeds	Di
202	Participant 1: Not in our context. There are no elder family member.	ACFP	Di
203	Parents give what is available. Because it is for children.	ACFP, Influence on CF feeding	Di
204	Parents give what is found. Ther is no elder family member.	ACFP, Influence on CF feeding	Di
205	There is no influence.	ACFP, Influence on CF feeding	Di
207	Participant 1: Foods that we call different, for example, local grain,	Variety of foods	Di
208	maize, beans, rice, and if available, eggs, milk, and butter,	Variety of foods	Di
209	then meat.	Variety of foods	Di
222	Participant 1: We do not know. There is nothing. No such	ASF feeding practices	Di
223	experience.	ASF feeding practices	Di
229	Participant 1: Now we are giving fermented flatbread made from	ACFP	Di
230	grins and what we found in the house. We are making and give	ACFP	Di
231	fermented flatbread with legume-based dish. We do not give	ACFP	Di
232	different food every day. It is one type. If it is available, the child	ACFP	Di
233	could eat different foods. If it is available, variety of food is better.	ACFP	Di
296	Participant 2: First, like oats, beans, barely, local grain, wheat, then	Type of CF the child feeds	Di
297	mix these and grind together. These are porridge and fluid food	Type of CF the child feeds	Di
298	(soup).	Type of CF the child feeds	Di
348	Participant 2: Uh... when legume-based dish, meat, or eggs are	ACFP	Di
349	eaten, it gives a lot of strength. The child’s body will be just fine.	ACFP	Di
350	When the child is cared for; the child grows up properly.	ACFP	Di
351	It is also, good if the child eats vegetables and fruits.	ACFP	Di
359	Participant 2: At 6 months, the child began to eat a teaspoon of	Amount of CF at a time	Di
360	porridge. Not with a big spoon, but with a small spoon.	Amount of CF at a time	Di
361	When the child is growing, bread will be given.	Amount of CF at a time	Di
376	Participant 2: Parents in our context feed children like we do.	Type of CF the child feeds, Variety of foods	Di
377	We feed children what we have. No milk, no meat, no	Type of CF the child feeds, Variety of foods	Di
378	vegetables. We simply feed porridge, or liquid food	Type of CF the child feeds, Variety of foods	Di
379	(soup-like liquid), or legume-based dish, like that. We feed	Type of CF the child feeds, Variety of foods	Di

Line No	Text	Codes	Synthesis1
380	children thick legume-based dish twice with a spoon.	Type of CF the child feeds, Variety of foods	Di
381	They pick up with their fingers and eat.	Type of CF the child feeds, Variety of foods	Di
401	Participant 2: There is meat nearby (butcher shop) but we do not	ASF feeding practices, Poor	Di
402	have it. But others who have resources rent milk and give for	ASF feeding practices, Poor	Di
432	children.		
433	Participant 2: Now, what we do for the child, it is porridge	Poor, Type of CF the child feeds	Di
434	and liquid food (soup-like liquid). Just it is since there is shortage of	Poor, Type of CF the child feeds	Di
435	resources.	Poor, Type of CF the child feeds	Di
436	Participant 2: There should have been different kinds of food.	Variety of foods	Di
439	Participant 2: If there is no financial problem, milk will be given in	Poor, Variety of foods	Di
440	the morning, meat, and eggs in the afternoon. We are giving	Poor, Variety of foods	Di
441	porridge at dinner time. The child eats and sleeps. If so, the child	Poor, Variety of foods	Di
442	does not suckle the breasts at night, and spends the night without	Poor, Variety of foods	Di
443	any problem.	Poor, Variety of foods	Di
495	Participant 3: Porridge and liquid food (soup-like liquid) made from	Type of CF the child feeds	Di
496	grains.	Type of CF the child feeds	Di
497	Participant 3: We prepared the porridge flour from beans, barley,	Ingredients of porridge	Di
498	red local grain, a little maize, and nuts.	Ingredients of porridge	Di
506	Participant 3: It is for porridge and liquid food (soup-like liquid).	ACFP, K. regarding CFP	Di
519	Participant 3: It is, as far as the child satisfied. We don't know the	Amount of CF at a time, K. regarding CFP	Di
520	amount, but feed until the child satisfied (refuses). At six months,	Amount of CF at a time, K. regarding CFP	Di
521	we were given 3 to 4 spoons with a small one.	Amount of CF at a time, K. regarding CFP	Di
524	Participant 3: It is different.	Type of CF the child feeds	Di
526	Participant 3: All the vegetables are enough. From home, it is	Variety of foods	Di
527	also, fermented flatbread and something like that in small amount.	Variety of foods	Di
528	But, since we can’t fulfill such, we give what is available.	Variety of foods	Di
571	Participant 3: It is being said to give animal source foods.	ASF feeding practices	Di
572	Animal source foods are needed but where do you get these?	ASF feeding practices, Poor	Di
573	Financial resources is required to get that.	ASF feeding practices, Poor	Di
577	Participant 3: Eggs, milk, and meat. If meat is available, it could be	Type of CF the child feeds	Di
578	grinded and given. We would give scrambled egg.	Type of CF the child feeds	Di

Line No	Text	Codes	Synthesis1
586	Participant 3: Regarding this [organ meats] …we do not know	ASF feeding practices	Di
587	(loughs). We have not used to eating organ meats (liver,	ASF feeding practices	Di
588	kidney, and heart) for the child. We never thought even to give	ASF feeding practices	Di
589	these things to the child.	ASF feeding practices	Di
605	Participant 3: We are making for the child just now is porridge	Type of CF the child feeds	Di
606	and liquid food (soup-like liquid). And the parent brings pasta and	Type of CF the child feeds	Di
607	pastina for the child. We give eggs once a month, not every day.	Type of CF the child feeds	Di
615	Participant 3: No vegetables. We will give nothing to the child but	Fru. & veg. feeding P.	Di
616	cabbage.	Fru. & veg. feeding P.	Di
675	Participant 4: We mix barley, wheat, oats, beans, chickpeas, rice,	Ingredients of porridge	Di
676	and red local grain.	Ingredients of porridge	Di
681	Participant 4: There are grains, cereals (pulses), eggs, and	ACFP	Di
682	how many things are brought from the urban area? We don't know	ACFP	Di
683	about that, but there are many parents who feed on this.	ACFP	Di
684	And we will also do our best.	ACFP	Di
690	Participant 4: For about two years, we gave CFs and we breast	Frequency of BF, Type of CF the child feeds	Co
691	feed as the child needs (whether the child is sleep or not, we also	Frequency of BF, Type of CF the child feeds	Co
692	make the child wakeup and breast feed).	Frequency of BF, Type of CF the child feeds	Co
695	After breastfeeding, complementary food is given. That is, it is	Frequency of BF, Type of CF the child feeds	Co
696	for up to two years. After two years, we can continue to feed	Type of CF the child feeds	Di
697	whatever we eat, but we can stop breastfeeding.	Type of CF the child feeds	Di
701	Participant 4: Now, the child feed porridge in the morning, and	Meal Frequency, Variety of foods	Di
	in the day, soup-like liquid made from grains flour.	Meal Frequency, Variety of foods	Di
702	At night, either milk or soup-like liquid is given.	Meal Frequency, Variety of foods	Di
703	Additionally, the child spent the night suckling the breast.	Meal Frequency, Variety of foods	Di
723	Participant 4: We have not tried eggs.	ASF feeding practices	Di
725	Participant 4: Well, we are not interested to it [eggs], i.e., bringing	ASF feeding practices	Di
726	eggs from market and giving it to the child on this day	ASF feeding practices	Di
727	[during fasting]? what it looks like…? The reason is that it is	ASF feeding practices	Di
728	difficult to buy from market and give it.	ASF feeding practices	Di

Line No	Text	Codes	Synthesis1
731	Participant 4: Because today [this period] is hot, it is	ASF feeding practices	Di
732	fasting, eggs can be collected [put for long time]. But eggs will be	ASF feeding practices	Di
733	useless after a week. So, it will not be used for the child [it will	ASF feeding practices	Di
734	spoil]. If it is from home, of course, that is not a problem. Since		
735	we do not have eggs at home; we did not try to buy and feed	ASF feeding practices	Di
736	the child.	ASF feeding practices	Di
740	Participant 4: Not yet started. We have not tried meat. Because	ASF feeding practices	Di
741	of the child's age (not old enough to eat meat), and now it	ASF feeding practices	Di
742	is fasting. We will not slaughter and feed meat at fasting time.	ASF feeding practices	Di
856	Participant 4: Milk, eggs, meat, if these things are given, the	ASF feeding practices, Type of CF the child feeds	Di
857	child will be healthy and strong.	ASF feeding practices, Type of CF the child feeds	Di
891	Participant 4: There is milk from home. We do not give milk	ASF feeding practices	Di
892	during the day because it does not go well with food. We will	ASF feeding practices	Di
893	boil one hand of milk with two hands of water, and we give it in a	ASF feeding practices	Di
	bottle at night either when the child cries or when wakes up from		
894	sleep.	ASF feeding practices	Di
917	Participant 4: After one year, the child will eat what the family eats,	Type of CF the child feeds	Di
	but what can we add to it? The child eats what the family eats,		
1046	Participant 5: It is good for the child if it is available with proteins	Type of CF the child feeds	Di
1047	such as cow's milk, meat, egg curd, and vitamins. From	Type of CF the child feeds	Di
1048	vegetables, cabbage, and Kosta, it is good for the body to drink	Type of CF the child feeds	Di
1049	juices such as orange, papaya, mango. From grain, wheat, local	Type of CF the child feeds	Di
1050	grain, and maize, prepared in the form of porridge flour.	Type of CF the child feeds	Di
1053	Participant 5: The amount of porridge prepared varies according to	Ingredients of porridge	Di
1054	the household's capacity (living situation). It is combining	Ingredients of porridge	Di
1055	all kinds of grains and cereals and grind together, as much	Ingredients of porridge	Di

Line No	Text	Codes	Synthesis1
1056	as possible (what the home produced).	Ingredients of porridge	Di
1092	A participant defined: Appropriate means giving the child what it	ACFP	Di
1093	wants, the right food.	ACFP	Di
1106	At 6 months to 8 months, the child should eat at least two to	Meal Frequency, Type of CF the child feeds	Di
1107	three spoons at a time. As the age increases, the amount of food	Meal Frequency, Type of CF the child feeds	Di
1108	increases. The type of food is also different. For example, if the	Meal Frequency, Type of CF the child feeds, Variety of foods	Di
1109	child eats bread at breakfast; it can be an orange or banana	Amount of CF at a time, Type of CF the child feeds	Di
1110	for a snack.	Meal Frequency, Type of CF the child feeds, Variety of foods	Di
1113	For a one-year-old and elder child, breakfast may be fermented	Meal Frequency, Type of CF the child feeds, Variety of foods	Di
1114	flatbread with legume-based dish in the morning or eats what the	Meal Frequency, Type of CF the child feeds, Variety of foods	Di
1115	house produces. At four o'clock, the snack may be bread or rice.	Type of CF the child feeds, Variety of foods	Di
1116	Parents prepare lunch in the same way as fermented flatbread,	Type of CF the child feeds, Variety of foods	Di
1117	add vegetables and give it for the child what it is nutritious.	Type of CF the child feeds, Variety of foods	Di
1118	At nine o'clock, a snack from orange or banana, whatever the	Type of CF the child feeds, Variety of foods	Di
1119	child can, so that the child does not get bored.	Type of CF the child feeds, Variety of foods	Di
1231	Participant 5: Eggs, milk, and meat.	ASF feeding practices	Di
1232	If possible, it is better to give eggs once every two days,	ASF feeding practices	Di
1233	and if not, once every three days so that the child does	ASF feeding practices	Di
1234	not get bored. Meat can be dried, grinded, and added to	ASF feeding practices	Di
1235	porridge or other food.	ASF feeding practices	Di
1387	Participant 6: In thick porridge.	Type of CF the child feeds	Di
1389	Participant 6: From wheat, red local grain, pulses, is added a	Ingredients of porridge	Di
1390	handful (small) of all grains and grinding together. Being made	Ingredients of porridge	Di
1391	from variety of food groups is being complete nutritious that	Ingredients of porridge	Di
1392	protects children from disease. Children will not be encouraged by	Ingredients of porridge	Di
1393	disease; they will able to walk faster if food is provided.	Ingredients of porridge	Di

Line No	Text	Codes	Synthesis1
1425	Participant 6: Balanced complementary food is what the house	ACFP	Di
1426	produced. There are eggs, there are just vegetables, there	ACFP	Di
1427	are vitamins, and again there is bread, pumpkin, these are	ACFP	Di
1428	appropriate food. Appropriate means made from different grains.	ACFP	Di
1432	Participant 6: Eggs, meat, beans, pumpkins, various fruits,	ACFP	Di
1433	disease-resistant vegetables. We know these as a farmer.	ACFP	Di
1434	Children should eat until they satisfied.	ACFP	Di
1449	The type of food is also appropriate. For example, if the child eats	Meal Frequency, Variety of foods	Di
1450	porridge in the morning, then in the afternoon, should have an	Meal Frequency, Variety of foods	Di
1451	egg or bread or should be different things.	Variety of foods	Di
1454	Childre should don't eat the same. The thickness of the porridge	Texture of porridge, Variety of foods	Di
1637	Participant 6: Parents do not hesitate to feed children what they	Type of CF the child feeds	Di
1638	have prepared at home. Because even if they don't bring it from	Type of CF the child feeds	Di
1639	market, even if they have a knowledge gaps; it is the community	K. regarding CFP, Type of CF the child feeds	Di
1640	practice that they feed that is available at home.	K. regarding CFP, Type of CF the child feeds	Di
1642	It is the community practice to feed what is available from home,	Type of CF the child feeds	Di
1643	such as carrots, red roots, tomatoes, eggs, and meat if	Type of CF the child feeds	Di
1644	available.	Type of CF the child feeds	Di
1698	Participant 7: Porridge.	Type of CF the child feeds	Di
1700	Participant 7: Is made from wheat, chickpeas, beans, peas, & oats.	Ingredients of porridge	Di
1720	Participant 7: It is giving balanced CF. If the child eats more, the	ACFP	Di
1721	child will become thin, distended abdomen. If it is	ACFP	Di
1722	given in proportion to the hour, the growth will increase and the	ACFP	Di
1723	appetite increases. Hourly means once in the morning, then at 4	ACFP	Di
1724	o'clock, 6 o'clock, 9 o'clock and 12 o'clock in the afternoon.	ACFP	Di
1728	Participant 7: Porridge in the morning, then at around four o'clock	ACFP	Di
1729	local legume-based dish. It is flat bread made from grain and	ACFP	Di
1730	legume-based dish, again in the evening, porridge will be given	ACFP	Di
1731	in the same way.	ACFP	Di
1737	Participant 7: Cabbage is made. Cabbage water. And that will be	Fru. & veg. feeding P.	Di
1738	done.	Fru. & veg. feeding P.	Di

Line No	Text	Codes	Synthesis1
1742	Participant 7: No, who knows in the rural area.	Fru. & veg. feeding P.	Di
1755	Participant 7: Uh… How much does that child eat? It's to eat small	Amount of CF at a time	Di
1756	amount. When they eat, it should be with a spoon.	Amount of CF at a time	Di
1759	Participant 7: Uh… We do not know. Laugh…	Amount of CF at a time	Di
1823	Participant 7: The boiled milk. Two cups of water and one cup of	ASF feeding practices, CF prohibition related	Di
1824	milk is made. After 6 months, two cups of water and one	ASF feeding practices, CF prohibition related	Di
1825	cup of milk is added and it will be boiled. Then, it will be given.	ASF feeding practices, CF prohibition related	Di
1841	Participant 7: Egg is given. Chicken; meat is given if available.	ASF feeding practices	Di
1845	Participant 7: Uh...no way. Not given.	ASF feeding practices	Di
1849	Participant 7: Of this, no, it is not normal (not given to children).	ASF feeding practices	Di
1967	Participant 8: For example, with Porridge.	Type of CF the child feeds	Di
1969	Participant 8: From grains. For example, it is made from oats,	Ingredients of porridge	Di
1970	barley, beans, peas, and chickpeas.	Ingredients of porridge	Di
1971	Preparing from different types, grinding it and giving as porridge.	Ingredients of porridge	Di
1974	Participant 8: If the child eats porridge in the morning, changing in	Amount of CF at a time	Di
1975	lunch time, like this.	Amount of CF at a time	Di
2001	Participant 8: It was explained earlier. That are complementary	ACFP	Di
2002	foods.	ACFP	Di
2005	Participant 8: Milk, meat, eggs, things from a shop (pasta,	Type of CF the child feeds	Di
2006	macaroni) such like these.	Type of CF the child feeds	Di
2028	Participant 8: Liquid food (soup-like liquid) is not too much.	Amount of CF at a time	Di
2029	A cup of porridge is enough. That will enough up to 6 o'clock, and	Amount of CF at a time	Di
2030	then it is time to change the food, it should be different from	Variety of foods	Di
2031	the food eaten in the morning or at six o'clock		
2032	If the child eats egg in the morning, at six o'clock better if it is	Variety of foods	Di
2033	macaroni or local legume-based dish. It is to give different foods.	Variety of foods	Di
2084	Participant 8: Milk, or meat (dried and ground) can be added to the	ASF feeding practices	Di

Line No	Text	Codes	Synthesis1
2085	porridge). Eggs are eaten in the form scrambled and boiled egg.	ASF feeding practices	Di
2185	A participant described: From variety of grain prepared, grinded	Type of CF the child feeds	Di
2186	and given in the form of slightly thin porridge.	Type of CF the child feeds	Di
2188	This participant further explained: There are grains in type such as	Ingredients of porridge	Di
2189	maize, red local grain, oats, barley, also peas or chickpeas, beans,	Ingredients of porridge	Di
2190	nuts, etc. They are mixed and grinded. Then, at the time of	CF preparation, Ingredients of porridge	Di
2191	Preparing	CF preparation, Ingredients of porridge	Di
2237	They also noted: Parents feed infants breast milk up to 6 months,	ACFP	Di
2238	as it was mentioned, after 6 months porridge flour will be prepared	ACFP	Di
2239	from variety of grains and cereals. Then it is given to the child in	ACFP	Di
2240	the form of porridge. It is appropriate when the child is given	ACFP	Di
2241	a diet based on animals, plants, vegetables, and fruits. Although	ACFP	Di
2242	complementary food starts at 6 months, parents are informed to	ACFP	Di
2243	continue breastfeeding at least until two years of child's age.	ACFP	Di
2250	Participant 9: At six months, if a baby eats two spoonsful of soft	Amount of CF at a time	Di
2251	porridge, that is enough. Therefore, it is for the composition,	Amount of CF at a time, Meal Frequency	Di
2253	for example, it means when it is composed of vegetables, fruits,	Variety of foods	Di
2254	animal source foods, and grains.	Variety of foods	Di
2264	Infants don't chew meat like adults do, but parents can dry, grind	ASF feeding practices	Di
2265	and mix it with the porridge. After potatoes are cooked, parents	ASF feeding practices	Di
2266	can feed to children by crushing it with their hands.	ASF feeding practices	Di
2327	Participant 9: If children are okay to eat, parents may find	ASF feeding practices	Di
2328	eggs from home. They can feed children in the form of scrambled	ASF feeding practices	Di
2329	eggs or mix eggs in the porridge during preparation.	ASF feeding practices	Di
2330	But children often do not eat as much as adults, so they are a little	ASF feeding practices	Di
2331	disturbance.	ASF feeding practices	Di
2336	Participant 9: Leave this, parents will not give. Some parents may	ASF feeding practices	Di
2337	dry the meat and mix with porridge. Other than this, they don't	ASF feeding practices	Di
2338	try liver, kidney, and heart meats for children. They do not give to	ASF feeding practices	Di
2339	children.	ASF feeding practices	Di
2341	Participant 9: If children in this age range eat eggs, milk, meat	ASF feeding practices	Di

Line No	Text	Codes	Synthesis1
2342	and liver and kidney, these are protein, children’s body will be built	ASF feeding practices	Di
2343	and good for health.	ASF feeding practices	Di
2511	A participant explained: It is simple, in the form of porridge. Apart	Type of CF the child feeds	Di
2512	from that, it can be a bolus of fermented flatbread with legume-	Type of CF the child feeds	Di
2513	based dish when the family eat food. Therefore, not only what the	Type of CF the child feeds	Di
2514	house produces but now, for example, if household has eggs, eggs	Type of CF the child feeds	Di
2515	will be mixed with porridge and given to the child.	Type of CF the child feeds	Di
2522	his participant listed: Maize, red local grain, barely, finger millet.	Ingredients of porridge	Di
2523	Three hands from grains and one hand from cereals. If parents	Ingredients of porridge	Di
2524	include local grain, barley, maize, sorghum, beans, and nuts, that is	Ingredients of porridge	Di
2525	good,	Ingredients of porridge	Di
2556	Participant 10: Appropriate complementary food means that the	ACFP	Di
2557	child's food is appropriate for the age. For example, it can be in the	ACFP	Di
2558	form of porridge or milk given at 6 months, and increase the	ACFP	Di
2559	thickness of porridge with age.	ACFP	Di
2568	Participant 10: The same food should not be given repeatedly so	Variety of foods	Di
2569	the child does not get bored. For example, if the child drank	Variety of foods	Di
2570	milk in the morning, it should be porridge at 6 o'clock and light	Variety of foods	Di
2571	baby food at night. But if the same food is given at different	Variety of foods	Di
2572	meal times; the child will get bored and not grow properly.	Variety of foods	Di
2573	This means that the child growth does not keep pace with age.	Variety of foods	Di
2631	Participant 10: Parents often give milk. They give eggs in	ASF feeding practices	Di
2632	the form of scrambled. But they don't give meat believing the meat	ASF feeding practices	Di

Line No	Text	Codes	Synthesis1
2633	will chock children and grinding takes time. Parents do not do that.	ASF feeding practices	Di
2634	If there is dried meat, they can grind and add it when they prepare	ASF feeding practices	Di
2635	porridge, but they do not pay attention to it. If available, it is better	ASF feeding practices	Di
2640	to feed the child liver or kidney meats. However, parents	ASF feeding practices	Di
2641	don't get these sources of food. No resource. Meat is available	ASF feeding practices	Di
2642	during annual festivals like Christmas and new year. But the	ASF feeding practices	Di
2643	community will not get it any other time.	ASF feeding practices	Di
2781	A participant described: Porridge. If it is soft like honeycomb, the	Type of CF the child feeds	Di
2782	child can eat it because it does not be heavy for the stomach. If it is	Type of CF the child feeds	Di
2783	given in this form; it means that the child's growth will be good.	Type of CF the child feeds	Di
2786	Participant 11: Porridge is made from grains and legumes. One	Ingredients of porridge	Di
2787	handful of cereal and three handfuls of grain seeds is prepared.	Ingredients of porridge	Di
2788	Grains can be local grain, maize, sorghum, barley, wheat. Peas,	Ingredients of porridge	Di
2789	beans, chickpeas, lentils, and nuts can be grouped as legumes.	Ingredients of porridge	Di
2790	These can only be given in the form of porridge.	Ingredients of porridge	Di
2824	Participant 11: Appropriate complementary food is what parents	ACFP	Di
2825	feed children made from different types of grains and legumes	ACFP	Di
2826	(pulses) alongside breastfeeding. And what we call inappropriate	ACFP	Di
2827	if parents do not feed scientifically based grains and legumes, and	ACFP	Di
2828	if not breastfeed at the right time, we call this inappropriate	ACFP	Di
2829	CF feeding.	ACFP	Di
2832	Participant 11: As it is mentioned before, CF is appropriate if it is	ACFP	Di
2833	prepared from variety of grains, seeds, legumes, and animal source	ACFP	Di
2834	foods (eggs, milk, dry, grind and put the meat in a bottle), added	ACFP	Di
2835	on porridge in small amount and given to the child. It is called	ACFP	Di
2836	appropriate complementary food.	ACFP	Di
2844	eight to twelve times a day, and one cup of porridge	Amount of CF at a time, Breastfeeding	Co
2865	Participant 11: Variety means, it can be a vegetable that can be	Variety of foods	Di
2866	vitamins, protein, carbohydrates (energy), it is good if given in	Variety of foods	Di
2867	accordance with the science. For example, from protein, eggs	Variety of foods	Di
2868	or meat can be given. Cabbage, oranges, etc. are considered as	Variety of foods	Di
2869	vitamins.	Variety of foods	Di

Line No	Text	Codes	Synthesis1
2940	This participant emphasized: It is very important to feed animal	ASF feeding practices	Di
2941	source foods for the baby. Of these, for example, milk shall not be	ASF feeding practices	Di
2942	drunk alone, but it is in the form of porridge. If parents have milk, it	ASF feeding practices	Di
2943	Is better to prepare the porridge with milk, and to give for children.	ASF feeding practices	Di
2944	Since children cannot eat the meat as it is, dry the meat, grind,	ASF feeding practices	Di
2945	and add in the porridge so that they can eat.	ASF feeding practices	Di
3154	A participant stated: It can be started as a soft porridge. It is made	Type of CF the child feeds	Di
3155	from different grains.	Type of CF the child feeds	Di
3157	Participant 12: It is prepared from three handfuls of grains seeds	Ingredients of porridge	Di
3158	and one handful of pulses, and then given in the form of	Ingredients of porridge	Di
3159	porridge. Grains like wheat, local grain, oats etc. And from	Ingredients of porridge	Di
3160	legumes, it means things like beans and chickpeas.	Ingredients of porridge	Di
3176	Participant 12: It is believed that these are food groups that parents	ACFP	Di
3177	prepare from their own hands (homes). What we call inappropriate	ACFP	Di
3178	means things that are packed and sold in shops like	ACFP	Di
3179	biscuits and candies are called inappropriate.	ACFP	Di
3191	lunch, and snacks. The older the child gets, the more food eats.	Meal Frequency, Variety of foods	Di
3208	Especially from 6 months to 9 months, parents may give one or	Amount of CF at a time	Di
3209	two small boluses of food when they eat.	Amount of CF at a time	Di
3246	Participant 12: There is a need to give priority to children such	ASF feeding practices	Di
3247	meat types like liver, heart, and kidney. If eggs and milk are	ASF feeding practices	Di
3248	available, it is better to give occasionally, not even	ASF feeding practices	Di
3249	continuously.	ASF feeding practices	Di
3253	Participant 12: Parents often have no idea that children need meat.	ASF feeding practices	Di
3254	There is a gap in this case. Particularly, there is a lack of	ASF feeding practices	Di
3255	understanding that liver, heart, and kidneys are important for	ASF feeding practices	Di
3256	children. Possibly it is giving meat what is found, that is giving	ASF feeding practices	Di
3257	something, the family foods. But nowadays, parents grind dried	ASF feeding practices	Di
3258	meat and add it to the porridge. Especially mothers give eggs.	ASF feeding practices	Di
3259	But it is not enough.	ASF feeding practices	Di
3438	Participant 13: The district is a grain producer. For example,	Ingredients of porridge	Di

Line No	Text	Codes	Synthesis1
3439	parents can use barley, beans, peas, chickpeas, sorghum, wheat,	Ingredients of porridge	Di
3440	finger millet, etc.	Ingredients of porridge	Di
3468	They also explained: Appropriate complementary foods are those	ACFP	Di
3469	that contain all the nutrients a child may need at this age.	ACFP	Di
3473	This participant continued: If the food contains all what it is call	ACFP	Di
3474	appropriate, such as vegetables, grains, and animal products such	ACFP	Di
3475	as eggs, cow's milk, it is a proper or complete complementary	ACFP	Di
3476	foods. It is better to continue breast feeding up to two years along	ACFP	Di
3477	with the complementary food.	ACFP	Di
3489	And the amount should be given as much as the child can take.	Amount of CF at a time, Meal Frequency	Di
3490	The variety (composition) should also include vegetables, fruits,	Amount of CF at a time, Variety of foods	Di
3491	animal products, legumes, and grains, according to what is	Variety of foods	Di
3492	mentioned earlier. As the age increases, the amount and	Variety of foods	Di
3493	frequency should also increase. But the composition is the same.	Amount of CF at a time, Meal Frequency, Variety of foods	Di
3586	There is no habit of thinking that even meat will benefit children	ASF feeding practices	Di
3587	apart from the family. As it is often known, meat is t the	ASF feeding practices	Di
3588	available at time of the festivals because the community is low	ASF feeding practices	Di
3589	income as a country. For example, meat is available during	ASF feeding practices	Di
3590	Christmas, Easter, and other annual holidays. At other times	ASF feeding practices	Di
3591	there is often no meat. Even when there is meat, the focus is on	ASF feeding practices	Di
3592	adults rather than giving priority to children. There is no practice of	ASF feeding practices	Di
3593	preparing and giving to children. Therefore, as it is mentioned it is	ASF feeding practices	Di
3594	essential to overcome this perception gaps by improving	ASF feeding practices	Di
3595	community awareness.	ASF feeding practices	Di
3596	The vicious circle will not stop. This means inappropriately fed	ASF feeding practices	Di
3597	children will also give malnourished children.	ASF feeding practices	Di
3598	also be malnourished, and the circle will continue and it will bring	ASF feeding practices stopped	Di
3599	pressure on the country's economy and social development.	ASF feeding practices	Di

Line No	Text	Codes	Synthesis1
3603	Participant 13: Cow's milk and eggs are often found in the	ASF feeding practices	Di
3604	community. If these can be given well and other food types such as	ASF feeding practices	Di
3605	grains, vegetables, fruits, it can be good and sufficient.	ASF feeding practices food	Di
3778	Participant 14: The first practice is in the form of porridge, which	Type of CF the child feeds	Di
3779	parents or guardians prepare from all kinds of grains.	Type of CF the child feeds	Di
3781	Participant 14: It is made from grains, fruits, vegetables, and seeds.	Ingredients of porridge	Di
3782	It is prepared in the form of porridge.	CF preparation, Ingredients of porridge	Di
3871	Participant 14: What we call a balanced diet; is food prepared for	ACFP	Di
3872	children after six months. When it is prepared, it is to make sure	ACFP	Di
3873	that different type of seeds, grains are put together and prepared	ACFP	Di
3874	accordingly. That means, first, its benefit is to prevent diseases.	ACFP	Di
3875	Second, children growth will be accelerated, they will be strong,	ACFP	Di
3876	and when they reach for education, their reception will be better.	ACFP	Di
3887	Participant 14: Yes, that is right. CF is given after 6 months	ACFP	Di
3888	according to children's age. There are CFs given at 6 to 8 months,	ACFP	Di
3889	8-12 months, 12-18 months, and 18-24 months. It is not just about	ACFP	Di
3890	continuing to give food. It is being done in addition to the mother's	ACFP	Di
3891	breast milk. The amount is in spoonful. It is the mother who can feed	ACFP, Texture of porridge	Di
3899	by spoonful. With this much, children will made to lick or take small	Amount of CF at a time, Meal Frequency	Di
3900	boluses of food.	Amount of CF at a time	Di
3905	Participant 14: As it was mentioned earlier, following 6 months of	Type of CF the child feeds	Di
3906	age different foods are prepared in the form of porridge for the first	Type of CF the child feeds	Di
3907	two to three months. But after that (from 8 months to 12 months)	Type of CF the child feeds	Di
3908	this is different. As children grow or increased in age, the amount	Type of CF the child feeds	Di
3909	of food varies in size. For example, a thin porridge is made while	Texture of porridge, Type of CF the child feeds	Di
3910	the content is the same up to one year old. This is up to one year	Texture of porridge, Type of CF the child feeds	Di
3911	old. After one year old, the complementary food will be thicker until	Texture of porridge, Type of CF the child feeds	Di
3914	the twenty-fourth month. The food that the family eats also given.	Texture of porridge, Type of CF the child feeds, Variety of foods	Di
3915	When that is done, breastfeeding should continue with 10-12 times a	Frequency of BF, Type of CF the child feeds, Variety of foods	Co

Line No	Text	Codes	Synthes1
3916	day. Because complementary foods are in addition to breast milk, it is	Breastfeeding, Frequency of BF, Type of CF the child feeds, Variety of foods	Co
3918	not totally giving up breast feeding. Many mothers now make the most	Breastfeeding, Type of CF the child feeds, Variety of foods	Co
3919	mistakes, which is to stop breastfeeding after introducing	Breastfeeding, Type of CF the child feeds, Variety of foods	Co
3920	complementary foods. They interrupt breast feeding. But this is very	Breastfeeding, Type of CF the child feeds, Variety of foods	Co
3921	forbidden and should not happen. Health workers are strongly	Breastfeeding, Type of CF the child feeds, Variety of foods	Co
3922	advising mothers not to do like this. This should not be happened.	Breastfeeding, Type of CF the child feeds, Variety of foods	Co
3923	Because complementary food is not considered sufficient mother’s	Breastfeeding, Type of CF the child feeds, Variety of foods	Co
3925	breast milk. It is very important until two years of age.	Breastfeeding, Type of CF the child feeds, Variety of foods	Co
3926	Generally, complementary feeding is like this.	Breastfeeding, Type of CF the child feeds, Variety of foods	Co
3931	Participant 14: These can be fruits, vegetables, milk seeds in	Type of CF the child feeds	Di
3932	between. Uh... that is a lot. For example, thick porridge is the form of	Type of CF the child feeds	Di
3933	porridge that the child has with his/her own plate.	Type of CF the child feeds	Di
3934	It is not talking about what the other person can eat. It is not	Type of CF the child feeds	Di
3935	possible to say we are feeding children complementary foods	Type of CF the child feeds	Di
3936	by giving family foods at lower age or the first 6, 7, 8, 9 months up to	Type of CF the child feeds	Di
3939	a year. It means there is a need to explain and teach parents in detail.	Type of CF the child feeds	Di
3945	Participant 14: Animal sources such as meat, eggs, milk, and fish if	ASF feeding practices	Di
3946	possible. When talking about this, it is the one that are	ACFP, ASF feeding practices	Di
3947	available in the house.	ACFP, ASF feeding practices	Di
4060	Participant 14: It is mainly milk and eggs are available in	ASF feeding practices	Di
4061	most households. Therefore, these should be given to children at	ASF feeding practices	Di
4062	breakfast, lunch, and dinner. Mothers may consider the benefits	ASF feeding practices	Di
4063	of cow milk as simple. But it is good if there is a way to feed what is	ASF feeding practices	Di
4064	available in the right way and at the right time.	ASF feeding practices, Meal Frequency	Di
4134	The convenient situation for our context. But it is not to conclude	ACFP, Foodstuffs related, Lack of attention to CFP, Mother’s current CFP	Ag
4135	that parents are feeding children using this convenient situation.	ACFP, Lack of attention to CFP, Mother’s current CFP	Att

Line No	Text	Codes	Synthesis1
4236	A participant stated: Mothers are counseled to prepare simple	Type of CF the child feeds	Di
4237	porridge flour as a mixture of nutritious, energy-giving, and vitamin	Type of CF the child feeds	Di
4238	(disease preventing) content.	Type of CF the child feeds	Di
4240	This participant added: Porridge flour can be prepared from different	Ingredients of porridge	Di
4241	types of grains with thorough washing, and drying, mixing, and	Ingredients of porridge	Di
4242	grinding after preparation. Parents will make a thin (medium texture)	Ingredients of porridge	Di
4243	porridge and feed children.	Ingredients of porridge	Di
4273	They also noted: If mothers prepare CFs at the family level and feed	ACFP	Di
4274	children in the form of milk, porridge, in addition to breast milk,	ACFP	Di
4275	hygienically prepared and feed in time, and if vitamin A capsules	ACFP	Di
4276	given after six months, if children’s weight and height is measured	ACFP	Di
4277	(If progress is monitored), we believe that they are getting	ACFP	Di
4278	appropriate complementary food.	ACFP	Di
4282	Participant 15: If proteins, carbohydrates (energy), and vitamins	ACFP	Di
4283	(disease preventing), vegetable content foods, and iodized salt	ACFP	Di
4284	(very important for growth) given, we call it an appropriate.	ACFP	Di
4285	Health workers provide counseling for mothers to balance and	ACFP	Di
4286	prepare these foods.	ACFP	Di
4302	increases depending on the situation. It is recommended that the	Texture of porridge, Variety of foods	Di
4303	child to use iodized salt, take vitamin A every 6 months, get	Variety of foods	Di
4304	vaccinations properly and complete it at one year and three months.	Variety of foods	Di

Synthesis 1: Workload

Line No	Text	Codes	Synthesis 1
	Document: Barriers of ACFP		
539	Participant 3: It's resource. Resource, and when we are busy	Lack of time, Poor	Economy (Poverty),
540	work (making the liquor, local beverage), we do not have time to	Lack of time, Poor	Economy (Poverty),
541	prepare CF for the baby, we leave it.	Lack of time, Poor	Economy (Poverty),
611	Participant 3: Oh, we are in a hurry. We are running out of time.	Lack of time, Responsive feeding	Frequency of C. fee
612	We are busy. We will take out the liquor (local beverage).	Lack of time, Responsive feeding	Frequency of C. fee
763	Participant 4: Number of jobs and lack of time. First, we are	Lack of time	Workload
764	farmers; we plough the farmland, then there are seedlings, once	Excessive workload, Lack of time	Workload
765	planted and grow, there is weeding, followed by harvesting.	Excessive workload, Lack of time	Workload
766	A farmer has no rest. We have cows. No child [elder child]	Excessive workload, Lack of time	Workload
767	to assist us. We are the one who rises and falls	Excessive workload, Lack of time	Workload
768	[we do everything] with no rest. We run together the cattle,	Excessive workload, Lack of time	Workload
769	draw [fetch] water from the well, and water the cows. The	Excessive workload, Lack of time	Workload
770	previous children are not grown like this child. we do not know	Excessive workload, Lack of time	Workload
771	before. We have tried for this child because this child is lucky.	Excessive workload, Lack of time	Workload
788	market for which not found at home, but there is lack of time.	CF preparation, Foodstuffs related, Lack of time	Agriculture product,
789	We have no resource problem. We produce barley, wheat,	CF preparation, Foodstuffs related, Lack of time	Agriculture product,
924	We are farmers. We will not be able to achieve this all.	CF prohibition related, Excessive workload, Lack of time	Misconceptions, Wor
925	Because there is a lot to these foods, a lot of science. It is not just	CF prohibition related, Excessive workload, Lack of time	Misconceptions, Wor
926	be cut and give. We do not have time because we are busy.	CF prohibition related, Excessive workload, Lack of time	Misconceptions, Wor
927	We have cattle, how many things we are doing to live?	CF prohibition related, Excessive workload, Lack of time	Misconceptions, Wor
950	if our house is full, it is not found simply, it is by working hard.	Excessive workload	Workload
951	Whether it is vegetables or anything, we just work throughout the	Excessive workload	Workload
952	day. This is the case, but there is scarcity of energy to do many	Excessive workload	Workload
953	things for the child from home. We lack energy, not lack of	Excessive workload, Lack of time	Workload
954	property. Therefore, nothing bothers us at home except we have	Excessive workload, Lack of time	Workload
955	no helper, lack of time and energy.	Excessive workload, Lack of time	Workload
1168	Participant 5: It is the Time. Parents see their time as a rare	Lack of time	Workload
1169	moment. Now is the time for cultivation, they don't have enough	Lack of time	Workload
1170	time for harvesting, farming.	Lack of time	Workload

Line No	Text	Codes	Synthesis 1
1172	For example, if mothers are asked why not providing CF in the	Lack of time, Reluctance	Attitude, Workload
1173	morning, they say we sweep the cow dung, and clean the house	Lack of time, Reluctance	Attitude, Workload
1174	at that time; they are not willing. They reply we will prepare	Lack of time, Reluctance	Attitude, Workload
1175	“When we sit down, or when we make coffee, it's enough”	Lack of time, Reluctance	Attitude, Workload
1176	After the child is very hungry, parents tried to feed by force.	Lack of time, Reluctance	Attitude, Workload
1177	Time is used for other work. They don't say that if we take care of	Lack of time, Reluctance	Attitude, Workload
1178	the children, they will reach at a good level.	Lack of time, Reluctance	Attitude, Workload
1179	They still say the child grows by opportunity.	Lack of time, Reluctance	Attitude, Workload
1288	Participant 5: Because there is lack of knowledge. Secondly,	K. regarding CFP, Lack of time	Knowledge, Workloa
1289	called the time. Lack of time.	Lack of time	Workload
1309	Participant 5: It is better if the focus is also on fathers in addition	Gender role, Men’s attitude to CFP	Attitude, Workload
1310	to mothers. Because counseling is mostly given for mothers.	Gender role, Men’s attitude to CFP	Attitude, Workload
1311	In this regard, males are not participatory. Dung also gives birth.	Gender role, Men’s attitude to CFP	Attitude, Workload
1312	This means; fathers do not have knowledge of child feeding.	Gender role, K. regarding CFP, Men’s attitude to CFP	Attitude, Workload
1313	It is just ended. Baby is born, the father says thank you and accept	Gender role, K. regarding CFP, Men’s attitude to CFP	Attitude, Workload
1314	the new born, but nothing about what does a child want?”	Gender role, K. regarding CFP, Men’s attitude to CFP	Attitude, Workload
1315	A father always wants the mother take care of the child, go out to	Gender role, K. regarding CFP, Men’s attitude to CFP	Attitude, Workload
1316	the work setting and work on, keeping the cattle.	Gender role, K. regarding CFP, Men’s attitude to CFP	Attitude, Workload
1317	There is no attention given for men parents regarding children.	Gender role, K. regarding CFP, Men’s attitude to CFP	Attitude, Knowledge,
1318	They say can’t be the farm land not being ploughed?	Gender role, Men’s attitude to CFP	Attitude, Workload
1319	How can we eat lunch unless we plough lands?	Gender role, Men’s attitude to CFP	Attitude, Workload
1320	It is better if attention is paid to men.	Gender role, Men’s attitude to CFP	Attitude, Workload

Line No	Text	Codes	Synthesis 1
1462	Participant 6: As a farmer, the obstacle to CF is that parents	Excessive workload, No rest	Workload
1463	want their work. It is urgent. It is a problem if one working day	Excessive workload, No rest	Workload
1464	goes. The obstacle is the work, but there is no other obstacle.	Excessive workload, Lack of time	Workload
1465	This means, lack of time. Get to work (hurry because work	Excessive workload, Lack of time	Workload
1466	make parents excited) and parents say, "give this food if elder	Excessive workload, Lack of time	Workload
1467	child is there" They go saying that "just a	Excessive workload, Lack of time	Workload
1468	lack of time." Time is gold.	Excessive workload, Lack of time	Workload
1773	Participant 7: Parents do not have time. In this regard, it is "Eat	Excessive workload, Lack of time	Workload
1774	what is cooked in the morning until they come back." The mother	Excessive workload, Lack of time	Workload
1775	goes to work telling the elder child "Give this." Mother does not	Excessive workload, Lack of time	Workload
1776	sit down and cook the food because of shortage of time.	Excessive workload, Lack of time	Workload
1878	Participant 7: Shortage of energy. Energy (lack of time).	Excessive workload, Lack of time	Workload
1879	Moreover, they have no knowledge. They have a lot of work.	Lack of time, Meal Frequency	Frequency of C. fee
2054	Second, lack of resource, however, parents provide the foods	Excessive workload	Workload
2055	available at home for children. It is the cost of living.	Excessive workload	Workload
2197	Participant 9: Family. Parents. But they are busy in work or	Decision maker of CF, Excessive workload, Foodstuffs related	Agriculture product,
2198	thinking that it will be difficult, everything [food items] is not	Decision maker of CF, Excessive workload, Foodstuffs related	Agriculture product,
2199	available at their home.	Decision maker of CF, Excessive workload, Foodstuffs related	Agriculture product,
2278	Participant 9: Lack of time is what most parents tell you. Of	Lack of time	Workload
2279	course, they have no rest. When they are focusing on work,	Excessive workload, Lack of time, No rest	Workload
2280	they did not do what they should do for children.	Excessive workload, Lack of time	Workload
2281	That is the reason why parents do not feed children as	Excessive workload, Lack of time	Workload
2282	needed. Otherwise, you will not find parents	Excessive workload, Lack of time	Workload
2283	who say we are not comfortable to feed children. But they have	Lack of time	Workload
2284	shortage of time. In terms of supply, there is shortage of	Attitude to change foodstuffs, Foodstuffs related, Lack of time	Agriculture product,
2285	supply. It is also a lack of attention and time or you will find	Attitude to CFP, Lack of time, Negligence	Attitude, Workload
2286	parents who say that we do not have enough time, we are	Attitude to CFP, Lack of time, Negligence	Attitude, Workload
2295	alone.	Lack of time, Negligence	Attitude, Workload

Line No	Text	Codes	Synthesis 1
2423	Participant 9: Why many parents do not give CF properly is	Lack of time	Workload
2424	the shortage of time. Second, by hearing it.	CF prohibition related, Lack of time	Misconceptions, Wor
2604	Apart from that, it will not be that much problem to feed what	Excessive workload, Poor	Economy (Poverty),
2605	is available at home. But lack of resource or poor family.	Excessive workload, Poor	Economy (Poverty),
2606	Apart from that, there is no as much a challenge to feed the	Excessive workload, Poor	Economy (Poverty),
2607	child. There may be workload. Parents reply that we won't	Excessive workload, Poor	Economy (Poverty),
2608	have time to prepare complementary food, for example, they	Excessive workload, Poor	Economy (Poverty),
2799	give an answer it will take our time, and it may be difficult	Lack of time, Reluctance	Attitude, Workload
2800	for us to bring it. is that they are often uneducated.	Lack of time, Reluctance	Attitude, Workload
2876	Another is shortage of time.	Lack of education, Lack of time	Knowledge, Workloa
2877	Since the parents work both outdoor and manage a large family,	Excessive workload, Gender role	Workload
2878	they do not give attention to prepare CF for children.	Excessive workload, Gender role	Workload
2879	Because they are busy, it can be a lack of time when they go	Excessive workload, Gender role, Lack of time	Workload
2880	outside, here and there. It means lack of attention. One, it takes	Excessive workload, Gender role, Lack of attention to CFP	Attitude, Workload
3169	time to prepare CF. They prefer to breastfeed, not to spent the	Lack of attention to CFP, Lack of time, Meal Frequency	Attitude, Frequency
3170	time in preparing CFs. On the other hand, there are parents	Lack of time, Meal Frequency	Frequency of C. fee
3171	who gives CFs. They are the ones who pay attention, &	Lack of time, Meal Frequency	Frequency of C. fee
3795	prepare. They take responsibility and feed children.	Decision maker of CF, Gender role	Time to start CF fee
3995	Participant 14: In terms of time, parents are often busy with	Excessive workload, Gender role, Lack of time	Workload
3996	Work. They have a lot of pressure. In this sense, they have	Excessive workload, Gender role, Lack of time	Workload
3997	fundamental problems. Parents especially are busy work	Excessive workload, Gender role, Lack of time	Workload
3998	(lack of time), is one of the key challenges of child feeding.	Excessive workload, Gender role, Lack of time	Workload
4079	First, parents don't have time. Especially during fasting time,	Lack of time	Workload
4080	they are not much to prepare food in advance for children.	Lack of time	Workload
4081	They treat children like adults. And if the child is breastfeeding	Lack of time	Workload
4082	they say no, breast is enough.	Lack of time	Workload
4321	Participant 15: Especially parents have workload. There is a lack	Excessive workload, Lack of attention to CFP	Attitude, Workload
4322	of attention to prepare in a way that suits children, understanding	Excessive workload, Lack of attention to CFP	Attitude, Workload
4323	problems (thinking that breast milk is enough).	Excessive workload, Lack of attention to CFP	Attitude, Workload
4418	work habits. Rural area parents have multiple jobs. They execute	Excessive workload	Workload
4419	both the in house and outdoor works in coordination. In this	Excessive workload	Workload
4420	sense, they may not pay attention to children. Because they do	Excessive workload	Workload
4421	many jobs at the family level, there are problems such as not	Excessive workload	Workload
4422	giving breast milk on time, not feeding CF on time even if the	Excessive workload	Workload
4423	food is prepared, and not feeding calmly (slowly).	Excessive workload	Workload

Synthesis 1: Misconceptions

Line No	Text	Codes	Synthesis 1
	Document: Barriers of ACFP		
164	Participant 1: If we had, we would not say that the child will grow	Child grows by opportunity, Poor	Economy (Pover
165	by opportunity. But since we do not have it, we just sit here and	Child grows by opportunity, Poor	Economy (Pover
166	say, "May the child be blessed to grow." If we had it, we would	Child grows by opportunity, Poor	Economy (Pover
167	give a lot. If we had, we would feed the child vegetables, eggs,	Child grows by opportunity, Poor	Economy (Pover
168	and milk. So, since we do not have it, the child eats what we eat.	Child grows by opportunity, Poor	Economy (Pover
184	Participant 1: If available, eggs and milk will be given to the	Feeding ASF on fasting days	Misconceptions
185	child. For those who say that "giving animal source foods	Feeding ASF on fasting days	Misconceptions
186	make us not fasting", it is to wash hands. Parents say “it smells	Feeding ASF on fasting days	Misconceptions
187	to us”, if we wash our hands, how it makes us not fasting.?	Feeding ASF on fasting days	Misconceptions
213	Participant 1: Yes, there are foods that are prohibited for	CF prohibition related	Misconceptions
214	children under 2 years of age. For example, it is said that it is not	CF prohibition related	Misconceptions
215	good to give potatoes. It is not comfortable to their stomach	CF prohibition related	Misconceptions
216	(saying it makes children sick). Other, it is also said that honey	CF prohibition related	Misconceptions
217	does not loosen their mouths. They do not open their mouth	CF prohibition related	Misconceptions
218	[cannot talk easily].	CF prohibition related	Misconceptions
280	Participant 2: Making the child to eat. Not to decrease the child’s	CF prohibition related, Time to start CF	Misconceptions,
281	appetite while grows up. If the child eats very well at 6 month,	CF prohibition related, Time to start CF	Misconceptions,
282	the appetite will decrease when	CF prohibition related, Time to start CF	Misconceptions,
283	grows up. That means if complementary food is given as infants	CF prohibition related, Time to start CF	Misconceptions,
284	need food first, then they will start to dislike food when age	CF prohibition related, Time to start CF	Misconceptions,
285	increases. We were informed to give CF when the infant is 6	CF prohibition related, Time to start CF	Misconceptions,
286	months old. But if you start CF late when the child’s age is	CF prohibition related, Time to start CF	Misconceptions,
287	at least 9 or 10 months old, the child will have no problem.	CF prohibition related, Time to start CF	Misconceptions,
288	They like it and their appetite will not decrease. Now, the child’s	CF prohibition related, Time to start CF	Misconceptions,
289	appetite is decreased. Now, the child’s appetite is decreased	CF prohibition related, Time to start CF	Misconceptions,
290	because the child started eating at 6 months old. In our opinion,	CF prohibition related, Time to start CF	Misconceptions,
291	if we had not started CF at 6 months old, the child would not have	CF prohibition related, Time to start CF	Misconceptions,
292	stopped eating now.	CF prohibition related, Time to start CF	Misconceptions,
387	Participant 2: For example, potatoes are not needed for a child.	CF prohibition related	Misconceptions
388	Not needed for a child. Since it becomes ascaris, they cannot eat.	CF prohibition related	Misconceptions
389	Otherwise, there is no problem if the child eats something else.	CF prohibition related	Misconceptions
390	It will be ascaris means, it will not comfortable for the child.	CF prohibition related	Misconceptions
391	Children can eat potatoes when they grow up.	CF prohibition related	Misconceptions

Line No	Text	Codes	Synthesis 1
392	Let alone children, even it is not for adults. So, we do not give	CF prohibition related	Misconceptions
393	potatoes for children.	CF prohibition related	Misconceptions
416	Participant 2: Yes, we give it to the child. On a fasting day, we	Feeding ASF on fasting days	Misconceptions
417	prepare the food separately with the child’s dish and feed. Then,	Feeding ASF on fasting days	Misconceptions
418	we wash our hands. Who says it smells/forces us not to fast,	Feeding ASF on fasting days	Misconceptions
419	we give it to the child, but we do not eat it. So, it does not be	Feeding ASF on fasting days	Misconceptions
420	obstacle to fasting.	Feeding ASF on fasting days	Misconceptions
562	Participant 3: Other persons told not give fermented flatbread	CF prohibition related	Misconceptions
563	with legume-based dish. But no other food type is prevented. The	CF prohibition related	Misconceptions
564	say, to forgive giving fermented flatbread with legume-based dish,	CF prohibition related	Misconceptions
565	and advise to give bread and other things. It is for strength, and	CF prohibition related	Misconceptions
566	to be fat. The child was a bit thin. And they told us to prepare and	CF prohibition related	Misconceptions
567	give bread and something like that. But we are giving fermented	CF prohibition related	Misconceptions
568	flatbread with legume-based dish.	CF prohibition related, Cup/bottle feeding	Food preparatio
591	Participant 3: Uh… we would have worked for the child if we had	Feeding ASF on fasting days, Poor	Economy (Pover
592	found it. But the absence of resoueces; What are you doing	Feeding ASF on fasting days, Poor	Economy (Pover
593	bought.? Regarding the meat, it is not its time (it is fasting). It will	Feeding ASF on fasting days, Poor	Economy (Pover
785	not be done for the child today. To make	Feeding ASF on fasting days, Foodstuffs related	Agriculture prod
829	Participant 4: We do not think that it should not be eaten, except	CF prohibition related	Misconceptions
830	for alcohol. It is alcohol. So, we do not have anything that is	CF prohibition related	Misconceptions
831	forbidden for a child. We think the child will eat properly if it is	CF prohibition related	Misconceptions
832	all available.	CF prohibition related	Misconceptions
835	Participant 4: Potato… we have not tried it. We will buy it for us	CF prohibition related	Misconceptions
836	and eat. But we did not try it for the child. In this context, elder	CF prohibition related	Misconceptions
837	family say that potato will be feces. Potatoes are not good	CF prohibition related	Misconceptions
838	because it increases the stool, so we never gave potato for the	CF prohibition related	Misconceptions
839	child. They, say potatoes are not good for baby; Firstly, it is not	CF prohibition related	Misconceptions
840	good for the stomach, and secondly, it is a stool (increases	CF prohibition related	Misconceptions
841	stool). We did not give that because we are studying what is said.	CF prohibition related	Misconceptions
842	Since we are studying what is meant by others, we did not give	CF prohibition related, Elders' advice	Misconceptions
843	it to the child. Except for potatoes or lack of capacity (resource),	CF prohibition related, Elders' advice	Misconceptions
844	there is nothing that can be said to be prohibited for children.	CF prohibition related, Elders' advice	Misconceptions
850	Participant 4: The child is defecating on the third day if we	CF prohibition related	Misconceptions
851	give it properly. In fact, the frequent thin stool [frequent, watery but	CF prohibition related	Misconceptions
852	small) stool stopped once the food is started. The child’s stool	CF prohibition related	Misconceptions

Line No	Text	Codes	Synthesis 1
853	did not increase.	CF prohibition related	Misconceptions
860	Participant 4: We may not get meat for the child today. There are	Feeding ASF on fasting days	Misconceptions
861	no eggs in the house now. But we are giving milk. We do not	Feeding ASF on fasting days	Misconceptions
862	believe that it forces us to breach fasting. If there is, we will	Feeding ASF on fasting days	Misconceptions
863	prepare child's food separately so that does not join us.	Feeding ASF on fasting days	Misconceptions
864	So, if utensils washed separately, what will happen to the parent?	Feeding ASF on fasting days	Misconceptions
865	If we do not eat it, why bother the parent?	Feeding ASF on fasting days	Misconceptions
876	Participant 4: The previous children grown up with their	Child grows by opportunity	Misconceptions
877	opportunity. But now this child is lucky. We have tried	Child grows by opportunity	Misconceptions
878	complementary foods for this child.	Child grows by opportunity	Misconceptions
895	One hand [cup] of milk is boiled together with two hands [cups] of	ASF feeding practices, CF prohibition related	Dietary diversity,
895	water	ASF feeding practices, CF prohibition related	Dietary diversity,
898	Participant 4: This is because we have been told. If you just boil the	CF prohibition related	Misconceptions
899	milk (without water) and pour it, the milk is too thick and it will	CF prohibition related	Misconceptions
900	not comfortable to the child. So, when it is boiled, two hands of water	CF prohibition related	Misconceptions
901	are added in one hand of milk.	CF prohibition related	Misconceptions
922	Participant 4: We did not start vegetables for the child. We have	CF prohibition related	Misconceptions
923	our own mango tree. But we did not try it. We are farmers.	CF prohibition related	Misconceptions
924	We will not be able to achieve this all. Because there is a lot to do	CF prohibition related, Excessive workload, Lack of time	Misconceptions,
925	for these foods, a lot of science. It is not just be cut and give.	CF prohibition related, Excessive workload, Lack of time	Misconceptions,
926	So, we do not have time. We do not have time because we are	CF prohibition related, Excessive workload, Lack of time	Misconceptions,
927	busy. We have cattle, how many things we do to live?	CF prohibition related, Excessive workload, Lack of time	Misconceptions,
928	Therefore, if you give anything [unhygienically], it will not make the	CF prohibition related, Excessive workload, Lack of time	Misconceptions,
929	child healthy. If you do not give it ready, it will not fit. So, we did not	CF prohibition related	Misconceptions
930	try it. We think it is "Better than being touched.	CF prohibition related	Misconceptions
934	We did not take care of the baby; we did not try to feed it.	CF prohibition related	Misconceptions
935	If the child has the experience of eating mango, and if we do not	CF prohibition related	Misconceptions
936	take care of him/her, the child will pick up and eat the it which	CF prohibition related, Cleanliness	Food preparatio
937	is fall on the ground, may be contaminated "It is better not to give	CF prohibition related, Cleanliness	Food preparatio
938	than to eat picking-up from the ground."	CF prohibition related, Cleanliness	Food preparatio
939	It is better for not to get used to feeding the mango than eating	CF prohibition related, Cleanliness	Food preparatio
940	by picking up from the ground. What bothers us….?	CF prohibition related, Cleanliness	Food preparatio
941	Banana is from the market so we did not try it.	CF prohibition related, Cleanliness	Food preparatio

Line No	Text	Codes	Synthesis 1
1216	Participant 5: We do not have prohibited food in this context. It is	CF prohibition related	Misconceptions
1217	all important. It is from attention and perspective, but there	CF prohibition related	Misconceptions
1218	is no saying that this is not necessary given for a child.	CF prohibition related	Misconceptions
1238	Participant 5: There is no such thing because they are children.	Feeding ASF on fasting days	Misconceptions
1239	There are many who say I do not prepare for the child, I smell	Feeding ASF on fasting days	Misconceptions
1240	it while the child is eating. There are some also prepare CF	Feeding ASF on fasting days	Misconceptions
1241	for the child. But most parents do not give animal source	Feeding ASF on fasting days	Misconceptions
1242	foods (meat or eggs) for children during fasting time.	Feeding ASF on fasting days	Misconceptions
1243	The problem is that, why do I smell it while the child is	Feeding ASF on fasting days	Misconceptions
1244	eating? Either I do not taste it? or they assume, I smell it	Feeding ASF on fasting days	Misconceptions
1245	and I will be as not fasting (tsome egedefalehu).	Feeding ASF on fasting days	Misconceptions
1246	Parents say the utensils will be contaminated, and we forget	Feeding ASF on fasting days	Misconceptions
1247	and touch the adult utensils. They have the mentality that we	Feeding ASF on fasting days	Misconceptions
1248	will be as not fasting due to smells. But they should not think like	Feeding ASF on fasting days	Misconceptions
1249	that, and the child can eat what is available i.e., whether it is	Feeding ASF on fasting days	Misconceptions
1250	meat, milk, or eggs.	Feeding ASF on fasting days	Misconceptions
1508	Participant 6: Food does not be prohibited. The child should eat	CF prohibition related	Misconceptions
1509	as until satisfied. There is no limit.	CF prohibition related	Misconceptions
1528	Participant 6: We counsel parents to separate the utensils, and	Feeding ASF on fasting days	Misconceptions
1529	let the child feed alone There are mothers who prepare and	Feeding ASF on fasting days	Misconceptions
1530	give it to children.	Feeding ASF on fasting days	Misconceptions
1570	A child grows up with opportunity is expected from elder relatives.	Child grows by opportunity	Misconceptions
1571	Some say, no, you take care of the child: A child not grows up	Child grows by opportunity	Misconceptions
1572	with opportunity, think for yourself. Do not think that a child grows	Child grows by opportunity	Misconceptions
1573	by opportunity. Some say we grew up like that. There is.	Child grows by opportunity	Misconceptions
1574	one year. They say that the stool will increase.	CF prohibition related, K. regarding CFP	Knowledge, Mis
1714	Participant 7: They say that we do not give food to children	CF prohibition related	Misconceptions
1715	because it makes defecation much. This is what they usually say	CF prohibition related	Misconceptions

Line No	Text	Codes	Synthesis 1
1716	in this context. They say, it makes diarrhea, makes	CF prohibition related	Misconceptions
1717	extra bowel, and they say it smells. They say that if the child uses	CF prohibition related	Misconceptions
1718	only breast, the feces will not smell.	CF prohibition related	Misconceptions
1812	A participant stated: They say that when the child eats eggs, it will	CF prohibition related	Misconceptions
1813	not be comfortable to his/her stomach.	CF prohibition related	Misconceptions
1816	his participant continued: Since the child does not eat too much, a	CF prohibition related, Mother's current CFP	Frequency of C.
1817	small scrambled egg will be given after 6 months. Not to eat much.	CF prohibition related, Mother's current CFP	Frequency of C.
1818	They say the stomach cannot bear the burden. It will be given in	CF prohibition related, Mother's current CFP	Frequency of C.
1819	small amount.	CF prohibition related, Mother's current CFP	Frequency of C.
1823	Participant 7: The boiled milk. Two cups of water and one cup of	ASF feeding practices, CF prohibition related	Dietary diversity,
1824	milk is made. After 6 months, two cups of water and one cup of	ASF feeding practices, CF prohibition related	Dietary diversity,
1825	milk is added and it will be boiled. Then, it will be given.	ASF feeding practices, CF prohibition related	Dietary diversity,
1828	Participant 7: Because it is hard. Because it contains butter in it.	CF prohibition related	Misconceptions
1829	It has butter. The milk is not separated from butter, because the	CF prohibition related	Misconceptions
1830	child cannot digest it.	CF prohibition related	Misconceptions
1833	Participant 7: When the child is growing up. But little water will be	CF prohibition related	Misconceptions
1834	added to the milk even if the child is growing up. A little water is	CF prohibition related	Misconceptions
1835	added on the milk even if child's age is above a year.	CF prohibition related	Misconceptions
1853	Participant 7: It is just to use children's utensils separately. But	Feeding ASF on fasting days	Misconceptions
1854	they say utensils will be contaminated. We don't give it [animal	Feeding ASF on fasting days	Misconceptions
1855	source foods] to children. They say, "eat legume-based dish" like	Feeding ASF on fasting days	Misconceptions
1856	the family eats. Parents say, 'children will touch us with their	Feeding ASF on fasting days	Misconceptions
1857	hands, the smell will make us as not fasting (breach fasting).	Feeding ASF on fasting days	Misconceptions
1870	Participant 7: Yes. The knowledge is mostly there. But there are	CF prohibition related, K. & practice mismatch	Attitude, Miscon
1871	some parents who only breastfeed.	CF prohibition related, K. & practice mismatch	Attitude, Miscon
1873	Participant 7: They do not give saying that "because it smells bad	CF prohibition related	Misconceptions
1874	and his stool gets too much."	CF prohibition related	Misconceptions
1991	Participant 8: Parents say this, children will defecate on our back;	CF prohibition related	Misconceptions

Line No	Text	Codes	Synthesis 1
1992	they defecate on our back when they eat complementary foods.	CF prohibition related	Misconceptions
1997	Participant 8: Oh! parents are saying that if something, like feces,	CF prohibition related	Misconceptions
1998	suddenly comes while they [parents] are walking. Holding a	CF prohibition related	Misconceptions
1999	previous practice. But now all is better.	CF prohibition related	Misconceptions
2096	Participant 8: Yes, parents say we are giving. Now time, milk is	Feeding ASF on fasting days	Misconceptions
2097	not available in most households, but if they get, they give milk.	Feeding ASF on fasting days	Misconceptions
2098	They do not say that it forces to breach fasting.	Feeding ASF on fasting days	Misconceptions
2209	Participant 9: The reason why parents say often confuses you. It	CF prohibition related	Misconceptions
2210	makes you laugh. Smile…. What do you think they will say...	CF prohibition related	Misconceptions
2211	“What if we start CFs at six months and what about children’s	CF prohibition related	Misconceptions
2212	feces”? You will find parents who say this. Laugh....	CF prohibition related	Misconceptions
2214	Participant 9: Children’s stools are abundant, smelly and parents	CF prohibition related	Misconceptions
2215	say “From now on? What about their stools...?” This means that	CF prohibition related	Misconceptions
2216	parents are disgusted to touch the children’s feces with hands.	CF prohibition related	Misconceptions
2217	Parents say “What if we sit in front of people?” You will face with	CF prohibition related	Misconceptions
2218	such things. But the local health workers tried to divert and	CF prohibition related	Misconceptions
2219	explain. Other than that, the main reason parents do not start at	CF prohibition related	Misconceptions
2220	6 months is lack of knowledge. Then, when they informed well,	CF prohibition related	Misconceptions
2221	they agreed and tried to prepare.	CF prohibition related	Misconceptions
2228	reason for this is what it was explained. Parents know that	CF prohibition related, Time to start CF	Misconceptions,
2229	complementary foods will be started at 6 months. But when they	CF prohibition related, Time to start CF	Misconceptions,
2230	are asked, “have you started?” they say no, we have not started,	CF prohibition related, Time to start CF	Misconceptions,
2231	sometimes we give milk, but we have not started porridge.	CF prohibition related, Time to start CF	Misconceptions,
2232	They say “from now on…?,the feces will smell, how can	CF prohibition related, Time to start CF	Misconceptions,
2233	we sit in front of people?.	CF prohibition related, Time to start CF	Misconceptions,

Line No	Text	Codes	Synthesis 1
2347	A participant shared: Since children do not fast like adults, it is	Feeding ASF on fasting days	Misconceptions
2348	important to give animal source foods even during fasting. But	Feeding ASF on fasting days	Misconceptions
2349	parents say that if it is fasting period, utensils will be contaminated.	Feeding ASF on fasting days	Misconceptions
2350	Many parents are disappointed because the smell is overwhelming	Feeding ASF on fasting days	Misconceptions
2351	(makes us as not fasting). They do not want to give. You also find	Feeding ASF on fasting days	Misconceptions
2352	some mothers get knowledge and give fasting foods using	Feeding ASF on fasting days	Misconceptions
2424	separate utensils even though it is a fasting time.	CF prohibition related, Lack of time	Misconceptions,
2425	appropriately is that shortage of time. Second, by hearing	CF prohibition related	Misconceptions
2426	a misconception. As it was explained, you find parents who say, if we	CF prohibition related	Misconceptions
2427	start CF early, the child's stool will increase.	CF prohibition related	Misconceptions
2818	Participant 10: To prepare CF, parents think that, it needs many	Can't implement ACFP	Misconceptions
2819	types, it is difficult, it will take a lot of time, and we have no time	Can't implement ACFP	Misconceptions
2820	to prepare. They get bored just thinking about it. Because of that, it	Can't implement ACFP	Misconceptions
2821	may happen that we don't prepare.	Boredom, Can't implement ACFP	Attitude, Miscon
2927	Participant 11: No, there is no such thing. Of course, there are	CF prohibition related	Misconceptions
2928	those who say what is wrong with soup-like liquid made from	CF prohibition related	Misconceptions
2929	grain flour. We faced with those who say that children should take	CF prohibition related	Misconceptions
2930	liquid food.	CF prohibition related	Misconceptions
2931	Participant 11: No, there is no such thing. Of course, there are	CF prohibition related, Texture of porridge	Food preparatio
2936	and a child who eats porridge does not urinate. Starting from	CF prohibition related, Texture of porridge	Food preparatio
2937	6 months, there is no food that a baby cannot eat.	CF prohibition related	Misconceptions
2957	Participant 11: On this... Some parents say that if you are fasting,	Feeding ASF on fasting days	Misconceptions
2958	you should not give your baby foods that are animal sources.	Feeding ASF on fasting days	Misconceptions
2959	Their reason is touch or contamination. They say it makes	Feeding ASF on fasting days	Misconceptions
2960	us as not fasting. There is a view that "When we give ASFs for a	Feeding ASF on fasting days	Misconceptions
2961	child, we will touch it, the object [utensils] will be contaminated.	Feeding ASF on fasting days	Misconceptions
2962	It is enough to make us as not fasting. There is something that	Feeding ASF on fasting days	Misconceptions
2963	has been overcome by teaching to prepare and give children's	Feeding ASF on fasting days	Misconceptions
2964	own food separately, and that there is nothing to make	Feeding ASF on fasting days	Misconceptions
2965	as not fasting. Though not completely solved.	Feeding ASF on fasting days	Misconceptions
3287	Participant 12: It is not common practice to offer foods of animal	Feeding ASF on fasting days	Misconceptions
3288	origin during fasting. They do not think that children should fast,	Feeding ASF on fasting days	Misconceptions

Line No	Text	Codes	Synthesis 1
3289	because it is not necessary for a child, but they think that we will	Feeding ASF on fasting days	Misconceptions
3290	be contaminated and it makes us as not fasting. Because parents	Feeding ASF on fasting days	Misconceptions
3291	are fasting, they may give other foods like porridge and other	Feeding ASF on fasting days	Misconceptions
3292	grain foods during the fasting period. During fasting period, meat	Feeding ASF on fasting days	Misconceptions
3293	(for example, chicken) is not given to children. Milk is given if	Feeding ASF on fasting days	Misconceptions
3294	if available, eggs are boiled and given if available.	Feeding ASF on fasting days	Misconceptions
3462	might not take the food that is prepared first. Second,	BM is enough, K. regarding CFP	Knowledge, Mis
3463	they think that breast milk is enough for the child. Third, the child’s	BM is enough, Lack of K. of nutritious foods	Knowledge, Mis
3528	digestive system may not be prepared to it. Fourth, it can also be	Opinion of child feeding after adults, Poor	Economy (Pover
3529	based on local practice. For example, children get food only after	Opinion of child feeding after adults	Misconceptions
3530	adults have eaten, according to the local child feeding practice.	Opinion of child feeding after adults	Misconceptions
3532	Again, there may be a problem of not giving the food properly,	Child grows by opportunity	Misconceptions
3533	not taking care of it, saying that a child "grows by chance".	Child grows by opportunity	Misconceptions
3534	Fifth, if the child is feed well, may become fat and fat and	Child grows by opportunity; Feeding well is making child fat	Misconceptions
3535	may a problem in the future.	Feeding well is making child fat	Misconceptions
3546	Participant 13: These problems are being solved now because	CF prohibition related	Misconceptions
3547	health workers are working on it. Previously, these things were	CF prohibition related	Misconceptions
3548	hard. For example, eggs, honey, such things are not necessary	CF prohibition related	Misconceptions
3549	for children, their intestines cannot handle it, their stomachs can't	CF prohibition related	Misconceptions
3550	digest it. But now, as the understanding is expanding, these	CF prohibition related	Misconceptions
3551	problems are being solved.	CF prohibition related	Misconceptions
3610	A participant observed: Considering our context, this is often	CF prohibition related	Misconceptions
3611	unusual. If animal is slaughtered at home, many times, meat	CF prohibition related	Misconceptions
3612	such as kidneys and liver are mostly used by the adult people.	CF prohibition related	Misconceptions
3613	Liver, kidney, and heart are good for children because they are	CF prohibition related	Misconceptions
3614	good for their health.	CF prohibition related	Misconceptions
3622	This participant added: In our context, it is difficult, as most of the	Feeding ASF on fasting days	Misconceptions
3623	community is fasting. So, it is a bit difficult to prepare and give	Feeding ASF on fasting days	Misconceptions
3624	these foods that are not eaten during fasting (food of animal	Feeding ASF on fasting days	Misconceptions
3625	origin). They don't give it. They say, utensils will be contaminated.	Feeding ASF on fasting days	Misconceptions
3626	There are attitudes that it contaminated us, the practice of	Feeding ASF on fasting days	Misconceptions
3627	preparing and giving during fasting is often low.	Feeding ASF on fasting days	Misconceptions

Line No	Text	Codes	Synthesis 1
3661	it is often tried to teach parents how to prepare it. But what could	CF prohibition related, Texture of porridge	Food preparatio
3662	not be corrected is that the child does not take it, it may be difficult	CF prohibition related, Texture of porridge	Food preparatio
3663	for the stomach or the intestine, which is why parents give thin	CF prohibition related, Texture of porridge	Food preparatio
3664	porridge to children. But if it is thin, the amount of water is too	CF prohibition related, Texture of porridge	Food preparatio
3665	much and it is useless. According to this, the porridge should be a	CF prohibition related, Texture of porridge	Food preparatio
3666	bit also be seen. This is not paying attention.	CF prohibition related, Texture of porridge	Food preparatio
3850	On other hand, there are many parents, in some places, who	CF prohibition related	Misconceptions
3851	believe that starting food earlier (at 6 months) will increase the	CF prohibition related	Misconceptions
3852	amount of their stools and their health will not be safe.	CF prohibition related	Misconceptions
3858	Participant 14: Feces smell different after eating CF and after	CF prohibition related	Misconceptions
3859	breastfeeding. Because the CF uses different food items, the	CF prohibition related	Misconceptions
3860	content changes, it has a smell, and again, they think that the	CF prohibition related	Misconceptions
3861	smell is mostly disliked by the community. Oh... and again, there	CF prohibition related	Misconceptions
3862	is a strong feeling in the community that we will be called that	CF prohibition related	Misconceptions
3863	“somebody’s child stool is smells like this from now on.” As a result,	CF prohibition related	Misconceptions
3864	parents do not start at six months with the view that children’s	CF prohibition related	Misconceptions
3865	feces will smell and increase [abundant]. There is a lack of	CF prohibition related	Misconceptions
3866	community awareness	CF prohibition related	Misconceptions
3951	are many things that the house produced. It is necessary to	CF prohibition related	Misconceptions
3952	explain clearly so that parents don't worry about what to milk,		
3953	grains, cereal seeds, and porridge, and don't give up thinking	CF prohibition related, K. regarding CFP	Knowledge, Mis
3954	that it won't be possible. It is necessary to teach about what is	CF prohibition related, K. regarding CFP	Knowledge, Mis
3955	available in the house enabling parents prepare and give in a	Can’t implement ACFP	Misconceptions
3956	proper manner. But it is not to buy.	Can’t implement ACFP	Misconceptions
3964	first is the lack of community awareness. Lack of awareness can	Can’t implement ACFP, K. regarding CFP	Knowledge, Mis
3966	be expressed in different ways. For example, if parents are not well	Can’t implement ACFP, K. regarding CFP	Knowledge, Mis
3967	informed, they may think about vegetables, grains, fruits, cereals,	Can’t implement ACFP, K. regarding CFP	Knowledge, Mis
3968	milk and milk products, and animal source foods, and they may say	Can’t implement ACFP, K. regarding CFP	Knowledge, Mis
3968	“We will not get overwhelmed” and leave what they have at home	Can’t implement ACFP, K. regarding CFP	Knowledge, Mis
3969	and look elsewhere. It is also expected to explain well so that they	Can’t implement ACFP, K. regarding CFP	Knowledge, Mis
3970	don't have the view that we can't afford it. They think we won't	Can’t implement ACFP, K. regarding CFP	Knowledge, Mis
3971	get it. That is the challenge (barrier).	Can’t implement ACFP, K. regarding CFP	Knowledge, Mis

Line No	Text	Codes	Synthesis 1
3974	There is a lot to say. Parents may perceive, if this is not	Can’t implement ACFP, K. regarding CFP	Knowledge, Mis
3975	available, without this, we cannot afford to buy these from	Can’t implement ACFP, K. regarding CFP	Knowledge, Mis
3976	market. So, we cannot do this and feel despair (hopelessness).	Can’t implement ACFP, K. regarding CFP	Knowledge, Mis
3977	The main challenge is lack of awareness. The problem of not	Can’t implement ACFP, K. regarding CFP	Knowledge, Mis
3978	taking care of children and not feeding is very apparent	Can’t implement ACFP, K. regarding CFP	Knowledge, Mis
3979	feeling that we have nothing, we cannot get what is required, or	Can’t implement ACFP	Misconceptions
3980	we cannot afford.	Can’t implement ACFP	Misconceptions
4000	Participant 14: In terms of income, as mentioned earlier,	Can’t implement ACFP, Poor	Economy (Pover
4001	if they are thinking about what to buy, they may not focus	Can’t implement ACFP, Poor	Economy (Pover
4002	on preparing and feeding CF because they do not have income.	Can’t implement ACFP, Poor	Economy (Pover
4003	It is one of the challenges that thinking of we do not have income.	Can’t implement ACFP, Poor	Economy (Pover
4045	Participant 14: Among the types of food in our context, food that is	CF prohibition related	Misconceptions
4046	not allowed to be given to children is not very common.	CF prohibition related	Misconceptions
4047	Unless there is a problem of providing the food items [diverse	CF prohibition related	Misconceptions
4048	foods], there is no community that would have any difficulty in	CF prohibition related	Misconceptions
4049	providing the available food used by the family.	CF prohibition related	Misconceptions
4073	Participant 14: There is a common problem with preparing and	Feeding ASF on fasting days	Misconceptions
4074	feeding foods of animal origin during fasting. This is very common.	Feeding ASF on fasting days	Misconceptions
4075	Especially if it is a fasting day (during fasting period), children	Feeding ASF on fasting days	Misconceptions
4076	may not consume ASFs.	Feeding ASF on fasting days	Misconceptions
4078	This problem exists too. Because it is a lack of awareness.	Feeding ASF on fasting days, K. regarding CFP	Knowledge, Mis
4085	They say, there is breast milk. Besides the breast, there is a	Feeding ASF on fasting days	Misconceptions
4086	perception that the child will eat when the hour of fasting (7 O’clock)	Feeding ASF on fasting days	Misconceptions
4087	reaches. They do not prepare and give animal source foods during	Feeding ASF on fasting days	Misconceptions
4088	fasting. What is the reason, it is fasting day (fasting period).	Feeding ASF on fasting days	Misconceptions
4089	Because the fasting practice is strong in this context, now they can	Feeding ASF on fasting days	Misconceptions
4090	give especially milk. It is not common to prepare eggs and give	Feeding ASF on fasting days	Misconceptions
4091	on fasting days. In connection with the fast, parents think that it	Feeding ASF on fasting days	Misconceptions
4092	makes us not fasting.	Feeding ASF on fasting days	Misconceptions
4094	And the practice of preparing egg and meat during fasting is that	Feeding ASF on fasting days	Misconceptions
4095	much very poor.	Feeding ASF on fasting days	Misconceptions
4110	To sum up, why can't parents properly follow an appropriate	Child grows by opportunity	Misconceptions
4111	complementary feeding method? (A child grows by chance or	Child grows by opportunity	Misconceptions
4112	opportunity).	Child grows by opportunity	Misconceptions

Line No	Text	Codes	Synthesis 1
4113	Participant 14: It is said that a child grows by opportunity	Child grows by opportunity	Misconceptions
4114	in some contexts. But it is not that much. But mainly, they don't	Child grows by opportunity	Misconceptions
4115	mind giving what they have. They think that there is	Child grows by opportunity	Misconceptions
4116	nothing better than a child. If it doesn't exist, it does not exist.	Child grows by opportunity	Misconceptions
4117	They may not think that we will find and give for the child.	Child grows by opportunity	Misconceptions
4118	They have the experience	Child grows by opportunity	Misconceptions
4358	Participant 15: According to our context, we have not received	CF prohibition related	Misconceptions
4359	any information about the presence of prohibited foods for	CF prohibition related	Misconceptions
4360	children from 6 months to 2 years. But maybe not starting CFs	CF prohibition related	Misconceptions
4361	early (6-8 months). Though the situation has changed now adays,	CF prohibition related	Misconceptions
4362	parents may not start complementary feeding for children less	CF prohibition related	Misconceptions
4363	than a year. The reason is a	CF prohibition related, K. regarding CFP	Knowledge, Mis
4366	problems do not start. But there is no situation where this food is	CF prohibition related, K. regarding CFP	Knowledge, Mis
4367	not needed for children.		
4381	Participant 15: If the animal source food is available in home, the	Feeding ASF on fasting days	Misconceptions
4382	community have no problem to prepare and give to children	Feeding ASF on fasting days	Misconceptions
4383	even if it is a fasting season. Milk and, eggs are prepared	Feeding ASF on fasting days	Misconceptions
4384	and given to children. The meat is the problem. The	Feeding ASF on fasting days	Misconceptions
4385	community does not have the practice of slaughtering and	Feeding ASF on fasting days	Misconceptions
4386	making chickens for children or getting meat during fasting.	Feeding ASF on fasting days	Misconceptions

Synthesis 1: Economy (Poverty)

Line No	Text	Codes	Synthesis 1
	Document: Barriers of ACFP		
146	Participant 1: It's the resource, but the knowledge is there.	K. regarding CFP, Poor	Economy (Poverty),
147	Health workers also tell us when we take the child	K. regarding CFP, Poor	Economy (Poverty),
148	for vaccine. We don't have, it's also because we are poor.	Poor	Economy (Poverty)
164	Participant 1: If we had, we would not say that the child will grow	Child grows by opportunity, Poor	Economy (Poverty),
165	up by opportunity. But since we do not have it, we will just sit	Child grows by opportunity, Poor	Economy (Poverty),
166	there and say, "May be blessed to grow." If we had it, we would	Child grows by opportunity, Poor	Economy (Poverty),
167	give it a lot. If we had, we would feed the child vegetables,	Child grows by opportunity, Poor	Economy (Poverty),
168	eggs, and milk. So, since we do not have it, the child eats what	Child grows by opportunity, Poor	Economy (Poverty),
169	the family eats.	Child grows by opportunity, Poor	Economy (Poverty),
172	Participant 1: Yes of course, but we don’t, have it?	Poor, The need of CF at 6-8m	Economy (Poverty),
176	Participant 1: If we have; milk, yogurt, butter, and eggs are	Poor, Type of CF the child feeds	Dietary diversity, Eco
177	foods of animal origin.	Poor, Type of CF the child feeds	Dietary diversity, Eco
180	Participant 1: We do not have the resource, so we will not give it	Poor	Economy (Poverty)
196	Participant 1: Well, if we get it, it is okay to prepare it. Now	Poor	Economy (Poverty)
197	is a good time and there is no work now. It is because we	Poor	Economy (Poverty)
198	don't have it.	Poor	Economy (Poverty)
368	Participant 2: We have no resource now. The child is suffering	Poor	Economy (Poverty)
369	because not eating properly. Therefore, if a job is opened	Poor	Economy (Poverty)
370	for us, we will do it and feed our children. Now there is	Poor	Economy (Poverty)
371	no resource to do this, but if we give well, the child will	Poor	Economy (Poverty)
401	Participant 2: There is meat nearby (butcher shop) but we don't	ASF feeding practices, Poor	Dietary diversity, Eco
402	have it. But the people who have resource rent milk and give	ASF feeding practices, Poor	Dietary diversity, Eco
403	to children.	ASF feeding practices, Poor	Dietary diversity, Eco
432	Participant 2: Now, what we do to our child is porridge	Poor, Type of CF the child feeds	Dietary diversity, Eco
433	and soup-like liquid made from grain flour. Just it is since	Poor, Type of CF the child feeds	Dietary diversity, Eco
434	there is shortage of resources.	Poor, Variety of foods	Dietary diversity, Eco
439	Participant 2: If we had... we would give the child milk in the	Poor, Variety of foods	Dietary diversity, Eco
440	morning, meat, and eggs in the afternoon. We used to give	Poor, Variety of foods	Dietary diversity, Eco
441	porridge for dinner. The child eats and sleep, does not suckle	Poor, Variety of foods	Dietary diversity, Eco
442	my breasts at night, and spends the night without any problem.	Poor, Variety of foods	Dietary diversity, Eco

Line No	Text	Codes	Synthesis 1
539	Participant 3: It's resource. resource, and when we are taking out	Lack of time, Poor	Economy (Poverty),
540	the liquor (local beverage), we don't have time to prepare CF for	Lack of time, Poor	Economy (Poverty),
541	the child, we leave it.	Lack of time, Poor	Economy (Poverty),
553	We were informed that to give these things for the child.	Info. access of information to IYCFP, Poor	Economy (Poverty),
554	But we say that we can't fulfil these things.	Info. access of information to IYCFP, Poor	Economy (Poverty),
555	Since the child now is increasing in age, we will give what we get.	Info. access of information to IYCFP, Poor	Economy (Poverty),
556	That is enough.	Info. access of information to IYCFP, Poor	Economy (Poverty),
572	Animal source foods are needed but where do you get these?	ASF feeding practices, Poor	Dietary diversity, Eco
573	Resource is required to get that.	ASF feeding practices, Poor	Dietary diversity, Eco
591	Participant 3: Uh… we would have prepared separately for the	Feeding ASF on fasting days, Poor	Economy (Poverty),
592	child if we had found it. But the absence of resources; What are	Feeding ASF on fasting days, Poor	Economy (Poverty),
593	you doing?	Feeding ASF on fasting days, Poor	Economy (Poverty),
1707	Participant 7: That's... laziness. Or who have no the capacity	Laziness, Poor	Attitude, Economy (P
1708	to prepare CF (poor). They may not be able to afford this.	Poor	Economy (Poverty)
1768	Participant 7: Lack of capacity. They may not have it. Now, if the	Poor	Economy (Poverty)
1769	pea and local grain are from the market, by what they will buy and	Poor	Economy (Poverty)
1770	prepare for children? That is why parents do not practice ACF.	Poor	Economy (Poverty)
1793	Participant 7: There is also a delivery [implementation] problem.	Foodstuffs related, Poor	Agriculture product,
1794	If it [flour] is maize alone, to be soup-like liquid or porridge, or to	Foodstuffs related, Poor	Agriculture product,
1795	be fermented flatbread, it does not obey [cannot come out from the	Foodstuffs related, Poor	Agriculture product,
1796	stove]. It will have no strength and it will be thin. But if there is	Foodstuffs related, Poor	Agriculture product,
1797	slight profit from hand [food items available at home], it is sold	Foodstuffs related, Poor	Agriculture product,
1798	and the vegetables and fruits that are found in the market	Foodstuffs related, Poor	Agriculture product,
1799	are bought and that is enough.	Foodstuffs related, Poor	Agriculture product,
2051	Participant 8: It will be lack of capability. If parents do not	Poor	Economy (Poverty)
2052	fulfil all things, they will probably give one from the food groups.	Poor	Economy (Poverty)
2595	Participant 10: There may be a supply problem. Farmers	Poor	Economy (Poverty)
2596	may not have land, or may be busy work. The types of grains	Poor	Economy (Poverty)
2597	needed to prepare complementary food may not be available	Poor	Economy (Poverty)
2598	at home. For example, parents want to make porridge, if there	Poor	Economy (Poverty)
2599	is barley and maize in her house, red local grain may not be	Poor	Economy (Poverty)
2600	available. There will be an increase in prices, for example, if they	Poor	Economy (Poverty)
2601	want to buy red local grain, if they do not have eggs at home,	Cost related issues, Poor	Economy (Poverty)
2602	there are such and such things [expensive]. Lack of resource,	Cost related issues	Economy (Poverty)
2603	that is it.	Cost related issues	Economy (Poverty)

Line No	Text	Codes	Synthesis 1
2604	Apart from that, it will not be that much of a problem for	Excessive workload, Poor	Economy (Poverty),
2605	children to eat what they have. But lack of resource or poor	Excessive workload, Poor	Economy (Poverty),
2606	family. There may be workload.	Excessive workload, Poor	Economy (Poverty),
2613	Parents know how to feed a child. Now, the first challenge is	Cost related issues, Negligence	Attitude, Economy (P
2614	the high cost of living, lack of attention, and negligence,	Cost related issues, Negligence	Attitude, Economy (P
2615	otherwise, they know that what they do for the child, how	Cost related issues, Negligence	Attitude, Economy (P
2616	the child will look better and gain weight.	Cost related issues, Negligence	Attitude, Economy (P
2620	Participant 10: First lack of resource (it is a little bit difficult to	Poor	Economy (Poverty)
2621	sell one food group from home and replace it with another).	Poor	Economy (Poverty)
2660	Parents may pay attention to children, but food groups may not		
2661	available, or there may be lack/ absence of resource. Families	Attitude to change foodstuffs, People’s opinion on ACFP, Poor	Attitude, Economy (P
2662	also want children to eat better and grow better. There will be	People’s opinion on ACFP, Poor	Attitude, Economy (P
2663	either absence of resource or supply problem.	People’s opinion on ACFP, Poor	Attitude, Economy (P
2890	Participant 11: In terms of price, some grains/legumes are now	Cost related issues	Economy (Poverty)
2891	very expensive. If parents don't have these food groups at home,	Cost related issues	Economy (Poverty)
2892	for example, peas, beans, grains, oil, they are all expensive.	Cost related issues	Economy (Poverty)
2893	There is no such thing as cheap, it may be difficult for parents to	Cost related issues	Economy (Poverty)
2894	prepare that [diverse meals]. Yes, there is a supply problem.	Cost related issues	Economy (Poverty)
2895	Parents go far from where they are and try to bring it, but they	Cost related issues	Economy (Poverty)
2896	don't find everything in the locality. It can be a variety of grains,	Cost related issues	Economy (Poverty)
2897	even vegetables, there are some parents who do not find	Cost related issues	Economy (Poverty)
2898	these things in the same locality.	Cost related issues	Economy (Poverty)
3234	Participant 12: In terms of price, of course, there are	CF preparation, Poor	Economy (Poverty),
3235	low-income people, but there is also the problem of	CF preparation, Negligence, Poor	Attitude, Economy (P
3236	preparing and feeding what is available at home. The product	CF preparation, Foodstuffs related, Negligence, Poor	Agriculture product,
3238	Because if they do not have, for example, legumes in home,	Attitude to change foodstuffs, Foodstuffs related, Poor	Agriculture product,
3523	Second, it could be the economy. When we say economy,	K. regarding CFP, Poor	Economy (Poverty),
3524	the household’s wealth that has at home or the crops they	Poor	Economy (Poverty)
3525	produced are important. It is a challenging. If they have not food	Poor	Economy (Poverty)
3526	groups in the house, they cannot prepare even if they have the	Poor	Economy (Poverty)
3527	knowledge. Thirdly, it can also be based on	Poor	Economy (Poverty)
4000	Participant 12: In terms of income, as mentioned earlier.	Can’t implement ACFP, Poor	Economy (Poverty),
4001	If parents are thinking about what to buy, they may not prepare	Can’t implement ACFP, Poor	Economy (Poverty),

Line No	Text	Codes	Synthesis 1
4002	CFs and feed because they do not have income.	Can’t implement ACFP, Poor	Economy (Poverty),
4003	Unless parents are well informed how to prepare CFs from food	Can’t implement ACFP, Poor	Economy (Poverty),
4004	groups available at home, believing that we do not have income	Can’t implement ACFP, Poor	Economy (Poverty),
4005	will be one of the challenges.	Can’t implement ACFP, Poor	Economy (Poverty),
4312	Participant 15: Economically poor parents. Parents may not get	Poor	Economy (Poverty)
4313	milk, meat, eggs. In this case, it is better to counsel to	Poor	Economy (Poverty)
4314	prepare CFs based on what they can afford. Now adays, there	Foodstuffs related, Poor	Agriculture product,
4315	is a scarcity of milk from time to time. There is no milk.	Foodstuffs related, Poor	Agriculture product,
4340	However, there are still many challenges, especially those	Poor	Economy (Poverty)
4341	with capacity problems, not paying attention (attitude	Lack of attention to CFP, Poor	Attitude, Economy (P
4342	problem) means giving only what is available at home, for	Lack of attention to CFP, Poor	Attitude, Economy (P

Synthesis 1: Community support for IYCFP

**Line No**	**Text**	**Codes**	**Synthesis 1**
	Document: Barriers of ACFP		
1606	Participant 6: It is nice to give birth and to kiss a baby.	Advice on BF/CF	Community support f
1607	This is convenient, it is possible. According to the healthcare	Advice on BF/CF	Community support f
1608	system, mothers	Advice on BF/CF, HF related, Info. access of information to IYCFP	Community support f
3074	Participant 11: Mothers are counseled to eat diverse diet not	Advice on BF/CF	Community support f
3075	only when she gives birth, but also from the day she says	Advice on BF/CF	Community support f
3076	“I am pregnant.” She is being told not only after birth, but also	Advice on BF/CF	Community support f
3077	before birth, she needs to get nutrition.	Advice on BF/CF	Community support f
3078	She will be informed that the fetus is feed through the	Advice on BF/CF	Community support f
3079	mediation of the placenta. After giving birth, the mother will be	Advice on BF/CF	Community support f
3080	advised to eat nutritious food and breastfeed well.	Advice on BF/CF	Community support f
3362	Participant 12: It is always a joy when a child comes into this	Advice on BF/CF	Community support f
3363	world. They (the family or community) are happy every	Advice on BF/CF	Community support f
3364	time when a mother gives birth. and the local health	Advice on BF/CF	Community support f
3365	workers visit their residences. This means the new mother will	Advice on BF/CF	Community support f
3366	be advised what to do after delivery. She is informed to fed	Advice on BF/CF	Community support f
3367	well, she is told when the child needs to start CF, she	Advice on BF/CF	Community support f
3368	monitored’ This represents the convenient situation.	Advice on BF/CF	Community support f
3712	Participant 13: The community considers a child as an asset.	Child is an asset	Community support f
3713	Because this child is born today. It is seen as an asset since	Child is an asset	Community support f
3714	parents thought that children will help when they grow up.	Child is an asset	Community support f
3715	So, what is expected from health workers is to fill their	Child is an asset	Community support f
3716	knowledge gaps. Since there are products, if knowledge gaps	Child is an asset	Community support f
3717	are solved and they develop positive attitudes toward hygienic	Child is an asset	Community support f
3718	preparation, and providing CFs, the community’s interest in	Child is an asset	Community support f

3719	child complementary feeding is said to be good.	Child is an asset	Community support f
3720	from that experience. In addition, as a mother gave birth;	Child is an asset	Community support f
4440	there is a situation where mothers, or mothers-in-law	Gifts to mother, Info. access of information to IYCFP	Community support f
4441	prepare many things and ask her.	Gifts to mother	Community support f
4442	Participant 15: In our context, there is a situation where	Gifts to mother	Community support f
4448	mothers, and the surrounding community ask the new mother.	Gifts to mother	Community support f
4449	When this happens, almost everyone in the village asked her	Gifts to mother	Community support f
4450	by giving something (what they have at home) starting from	Gifts to mother	Community support f
4451	milk.	Gifts to mother	Community support f
4452	The reason for this is that the mother has been bleed for	Advice on BF/CF, Gifts to mother	Community support f
4454	various reasons, and the community takes care of her at a	Advice on BF/CF, Gifts to mother	Community support f
4455	level that is said to be good enough to replace the lost blood	Advice on BF/CF, Gifts to mother	Community support f
4456	for fifteen days or more.	Advice on BF/CF, Gifts to mother	Community support f
4457	This is especially important for helping the mother begin	Advice on BF/CF, Gifts to mother	Community support f
4458	producing better-quality breast milk for the baby. In addition,	Advice on BF/CF, Gifts to mother	Community support f
4459	It is important in replacing the food lost during child birth and	Advice on BF/CF, Gifts to mother	Community support f
4460	for other reasons, has significant role in rebuilding her body,	Advice on BF/CF, Gifts to mother	Community support f
4461	returning it to its previous state, and supporting sufficient	Advice on BF/CF, Gifts to mother	Community support f
4462	breast milk production.	Advice on BF/CF, Gifts to mother	Community support f

Synthesis 1: Network of the health care system

Line No	Text	Codes	Synthesis 1
	Document: Barriers of ACFP		
552	Participant 3: There is a healthcare facility near in our context.	HF related, Info. access of information to IYCFP	Network of t
553	Health workers informed us to give the child these & theses	Info. access of information to IYCFP, Poor	Economy (
554	[CFS]. But we can't fulfill these things. Since the child now is	Info. access of information to IYCFP, Poor	Economy (
555	increasing in age, we are giving what we get. That is enough.	Info. access of information to IYCFP, Poor	Economy (
959	Participant 4: The first place where we can get information	Info. access of information to IYCFP	Network of t
960	is that, when we visit this health care facility monthly for ante	HF related, Info. access of information to IYCFP	Network of t
961	natal care. There is advice at these times. Since there is	HF related, Info. access of information to IYCFP	Network of t
962	advice, we call this a favorable situation to do what we are told to	HF related, Info. access of information to IYCFP	Network of t
963	do. But we did not visit this facility before (in previous years).	HF related, Info. access of information to IYCFP	Network of t
964	It is not the same as now. But now, we are monitored just the	HF related, Info. access of information to IYCFP	Network of t
965	start of the pregnancy, we are given lessons, and we try our best	Info. access of information to IYCFP	Network of t
966	to implement the lessons, we learned.	Info. access of information to IYCFP	Network of t
971	Participant 4: When we give birth here, health workers	HF related, Info. access of information to IYCFP	Network of t
972	teach us to breastfeed for six months, and then to start	HF related, Info. access of information to IYCFP	Network of t
973	complementary foods. We do that too. During vaccination, they	HF related, Info. access of information to IYCFP	Network of t
974	teach us not to work hard, to eat nutritious food, and they teach	Info. access of information to IYCFP	Network of t
975	everything. We will also try our best.	Info. access of information to IYCFP	Network of t
1300	Participant 5: They [health workers] respect for children.	HF related	Network of t
1301	The healthcare system is also complete. Everything is there.	HF related	Network of t
1302	While there are such conveniences, the problem lies in	HF related	Network of t
1303	implementation.	HF related	Network of t
1609	Regarding the health care system, mothers receive prenatal care	HF related	Network of t
1610	from 3 months of conception to 9 months until the child is born.	Advice on BF/CF, HF related, Info. access of information IYCFP	Community
1611	In addition, it is recommended by the government to have anemia	HF related, Info. access of information to IYCFP	Network of t
1612	pills and check-ups for fetus. Second, they go to the waiting	HF related, Info. access of information to IYCFP	Network of t
1613	room and give birth. This is a convenient thing.	HF related, Info. access of information to IYCFP	Network of t
1614	Education will be given to enable feeding appropriate food.	HF related, Info. access of information to IYCFP	Network of t

Line No	Text	Codes	Synthesis 1
1616	Advises is given, like keeping clothes clean. When women	HF related, Info. access of information to IYCFP	Network of t
1617	give birth in a health facility, the government provides breakfast,	HF related, Info. access of information to IYCFP	Network of t
1618	lunch, and dinner. This support is facilitated by the government so	HF related, Info. access of information to IYCFP	Network of t
1619	that mothers are free from work-related stress, allowing to	HF related, Info. access of information to IYCFP	Network of t
1620	rest and deliver safely. In in addition, health workers monitor	HF related, Info. access of information to IYCFP	Network of t
1621	throughout the day and give advice. Health workers also visit	HF related, Info. access of information to IYCFP	Network of t
1622	their residences and advise not to give birth at home.	HF related, Info. access of information to IYCFP	Network of t
1623	After the baby is born, the infant received vaccines, the mother is	HF related, Info. access of information to IYCFP	Network of t
1624	counseled to put on socks, hat and layered closes so that	HF related, Info. access of information to IYCFP	Network of t
1625	the child does not lose body heat or affected by external cold.	Info. access of information to IYCFP	Network of t
1626	Regarding nutrition, up to 6 months of age, the mother is advised	Info. access of information to IYCFP	Network of t
1627	to exclusively breastfed.	Info. access of information to IYCFP	Network of t
1917	Participant 7: Health workers informing mothers from which	HF related, HP related, Info. access of information to IYCFP	Network of t
1918	food groups they prepare and feed children.	HF related, HP related, Info. access of information to IYCFP	Network of t
1919	It is to prepare and provide that in a proper manner.	HF related, HP related, Info. access of information to IYCFP	Network of t
2453	Participant 9: Now when a baby is born, the family wants it.	Spacing birth interval	Network of t
2454	This is because now the time is changed. In the past, children	Spacing birth interval	Network of t
2455	were born without parents' planning, but now because there is	Spacing birth interval	Network of t
2456	birth control, parents give birth only when they want to.	Spacing birth interval	Network of t
2457	That's why they want children so much, they plan for, and want	Spacing birth interval	Network of t
2458	children to grow up well and live comfortably.	Spacing birth interval	Network of t
2680	Parents are also advised starting from babies begin vaccination,	Info. access of information to IYCFP	Network of t
2681	not to give any food for up to 6 months of age.	Info. access of information to IYCFP	Network of t
2682	Then, at 6 months, when they come for vitamin A, they are asked	Info. access of information to IYCFP	Network of t
2683	whether CF has started, and they might say" we are preparing	Info. access of information to IYCFP	Network of t
2684	or say we have started.	Info. access of information to IYCFP	Network of t
2685	Parents also discuss with development teams.	Info. access of information to IYCFP	Network of t
2708	They know that porridge is important for children under two years	Info. access of information to IYCFP	Network of t
2709	old. If they have any knowledge gaps; they can consultant	Info. access of information to IYCFP	Network of t
2710	health workers.	Info. access of information to IYCFP	Network of t
2711	There are also porridge preparation demonstrations for pregnant	Info. access of information to IYCFP	Network of t
2712	mothers and mothers who have children under two years old.	Info. access of information to IYCFP	Network of t
2713	During demonstrations, parents bring flour from their houses,	Info. access of information to IYCFP	Network of t
2714	when different flours are mixed; it becomes a balanced flour. In	Info. access of information to IYCFP	Network of t
2715	that way, demonstration is conducted by health workers.	Info. access of information to IYCFP	Network of t
2716	The demonstration includes; how much children should eat when	Info. access of information to IYCFP	Network of t
2717	porridge is offered like this.	Info. access of information to IYCFP	Network of t

Line No	Text	Codes	Synthesis 1
2725	Participant 10: By the way, now, when a baby is born it is by plan,	Spacing birth interval	Network of t
2726	and parents have no problem in take caring. For example,	Spacing birth interval	Network of t
2727	if the mother is not at home, the father will bring the child for	Spacing birth interval	Network of t
2728	vaccination. They have no problem to care of their child.	Spacing birth interval	Network of t
3352	Education is provided at the kebele level, and porridge	Foodstuffs related, HP related, Info. access of information to IYCFP	Agriculture
3353	preparation demonstrations are held three or four times per year.	HP related, Info. access of information to IYCFP	Network of t
3354	Since education is provided both during pregnancy and after	HP related, Info. access of information to IYCFP	Network of t
3355	childbirth, it creates a convenient situation for mothers. They can	HP related, Info. access of information to IYCFP	Network of t
3356	access support and advice nearby.	HP related, Info. access of information to IYCFP	Network of t
3446	parents can ask health workers and feed	HP related, Info. access of information to IYCFP	Network of t
3447	the child based on the knowledge they have acquired.	HP related, Info. access of information to IYCFP	Network of t
3556	Participant 13: Health workers conduct porridge cooking	Info. access of information to IYCFP	Network of t
3557	demonstrations for parents of young children and pregnant women	Info. access of information to IYCFP	Network of t
3558	involving development army groups.	Info. access of information to IYCFP	Network of t
4140	Participant 14: The problem of nutrition is the key problem of our	HF related, HP related	Network of t
4141	community. In order to overcome this, the health institutions have	HF related, HP related	Network of t
4142	structure from the top of the region to the kebele levels.	HF related, HP related	Network of t
4143	There are the district health offices, health centers, then there	HF related, HP related	Network of t
4144	are the health posts, and then there are villages, the	HF related, HP related	Network of t
4145	neighborhood, and the community.	HF related, HP related	Network of t
4146	It has a structure that enables the government	HF related, HP related	Network of t
4147	to achieve healthcare activities. The health office has	HF related, HP related	Network of t
4148	interconnected (hierarchical) activities with health facilities,	HF related, HP related	Network of t
4149	down to the kebeles. The upper structure enables the lower	HF related, HP related	Network of t
4150	structures. For example, health workers help women	HF related, HP related	Network of t
4151	development army members and give jobs. One enables	HF related, HP related	Network of t
4152	the other, creates awareness. In this way, there is a structure	HF related, HP related	Network of t
4159	through which health education messages reach to community.	HF related, HP related	Network of t
4160	A mother receives advice from health workers what to do	HF related, HP related	Network of t
4161	starting from conception till birth. A pregnant woman also receives	HF related, HP related	Network of t
4162	medical care.	HP related, Info. access of information to IYCFP	Network of t

Line No	Text	Codes	Synthesis 1
4163	After giving birth, the mother will be counseled about her and the	HP related, Info. access of information to IYCFP	Network of t
4164	child's health. For example, she informed to breastfeed exclusively	HP related, Info. access of information to IYCFP	Network of t
4165	up to 6 months in demand (at least 10-12 times in 24 hours).	HP related, Info. access of information to IYCFP	Network of t
4166	During vaccination, they advised to start complementary feeding	HP related, Info. access of information to IYCFP	Network of t
4167	after 6 months. In general, health worker’s support does not	HP related, Info. access of information to IYCFP	Network of t
4168	separate from her until the child aged two years.	HP related, Info. access of information to IYCFP	Network of t
4330	A participant reported: Porridge cooking demonstrations conducted	Info. access of information to IYCFP	Network of t
4331	at kebele levels, involving development army members,	Info. access of information to IYCFP	Network of t
4332	mothers with children, religious leaders, and youth. These	Info. access of information to IYCFP	Network of t
4333	activities aim to improve knowledge, build experience, and develop	Info. access of information to IYCFP	Network of t
4334	skills about complementary feeding.	Info. access of information to IYCFP	Network of t
4335	During cooking demonstration, mothers bring what they have at	Info. access of information to IYCFP	Network of t
4336	home such as vegetables, flour, beans, or peas etc.	Info. access of information to IYCFP	Network of t
4337	Pregnant mothers, mothers with children under 5 years old, and	Info. access of information to IYCFP	Network of t
4338	religious leaders are participated. So that	Info. access of information to IYCFP	Network of t
4339	mothers can learn from that experience.		
