# Supplementary material for: Appropriate complementary feeding practices and associated factors among mothers with infants aged 6–8 months in West Gojjam Zone, Northwest Ethiopia: A mixed methods study
Source: PLoS One. 2025 Oct 16;20(10):e0334740. doi: 10.1371/journal.pone.0334740 (PMC12530518; doi:10.1371/journal.pone.0334740)
Supplement: Attachment — Submitted filename: Manuscript Number - Feeding practice.docx [file pone.0334740.s004.docx]

**Manuscript Number**: PONE-D-24-46708

**Article Type:** Research Article

**Full Title:** Appropriate complementary feeding practices and associated factors among mothers

with infants aged 6–8 months in the West Gojjam Zone, Northwest Ethiopia: A mixed

methods study.

Reviewer: Luqman Abass

**1. Originality of the Research**

The study presents original research addressing appropriate complementary feeding practices (ACFP) among mothers of infants aged 6–8 months in the West Gojjam Zone, Northwest Ethiopia. The authors utilize both quantitative and qualitative methods to explore the prevalence and factors associated with ACFP, which is valuable given the existing gaps in literature on complementary feeding practices in rural Ethiopia.

**2. Novelty of the Results**

The results are new, particularly the use of the revised WHO 2021 IYCF indicators in assessing complementary feeding practices. This is a notable advancement over previous studies that used the older 2008 WHO indicators. Furthermore, the inclusion of both qualitative and quantitative data provides a deeper understanding of the barriers to ACFP in this specific rural Ethiopian context, contributing to the literature with new insights about the psychological, economic, and cultural factors influencing feeding practices.

**3. Technical Rigor of the Study**

- **Methods:** The study uses a robust mixed-methods design, with a large sample size (802 mothers). The data collection methods are well-structured, using validated instruments adapted from the Ethiopia Mini-DHS and revised WHO IYCF indicators. The analysis is appropriate, including multivariable logistic regression for identifying factors associated with ACFP and thematic analysis for the qualitative data.
- **Statistics:** The use of Epi-Info for sample size calculation and SPSS for statistical analysis ensures a high technical standard. The methods for testing multicollinearity, model fitness (Hosmer-Lemeshow), and handling the cluster sampling design are well-executed, with careful attention to statistical assumptions.

**4. Clarity of Reporting and Data Presentation**

- The article is generally well-organized, with clear sections outlining the background, methodology, results, and discussion. Tables and figures are used effectively to present key findings (e.g., Tables 4 and 5 present a comprehensive breakdown of complementary feeding practices and factors associated with ACFP).
- However, the manuscript could benefit from more detailed descriptions of some of the statistical procedures, such as the generalized linear mixed model, as this might not be familiar to all readers. The inclusion of more details on how the wealth index was calculated would enhance transparency in the methods.

**5. Conclusion and Data Support**

- The conclusions drawn are appropriate, based on the data presented. The study emphasizes the importance of postnatal complementary feeding counseling, addressing misconceptions, and improving household economic status. These conclusions are well-supported by the data, particularly from the multivariable logistic regression and qualitative findings.
- The authors recommend practical interventions, including strengthening postnatal counseling and addressing economic and cultural barriers, which aligns with the study's findings.

**6. Ethical Standards**

- The study complies with ethical standards, having received approval from the Institutional Review Board at Bahir Dar University. Written informed consent was obtained, and confidentiality was maintained throughout the data collection process.
- Ethical challenges such as ensuring voluntary participation and obtaining informed consent from illiterate mothers (using fingerprints) are adequately addressed.

**7. Adherence to Reporting Guidelines**

- The article generally adheres to standard reporting guidelines, including those for ethical conduct, informed consent, and transparency in data analysis. However, further clarification on the data availability statement and adherence to data-sharing community standards (e.g., deposition in a public repository) could strengthen compliance with open data practices.

**8. Language and Presentation**

- The manuscript is written in clear, standard English with minimal grammatical errors. However, there are occasional awkward phrasings (e.g., "flesh foods" could be more clearly stated as "animal-source foods"). These minor issues do not significantly detract from the overall clarity but could be addressed to improve readability.
- The manuscript could be improved by breaking up longer paragraphs, particularly in the discussion section, to enhance readability and clarity.
