# Supplementary material for: Appropriate complementary feeding practices and associated factors among mothers with infants aged 6–8 months in West Gojjam Zone, Northwest Ethiopia: A mixed methods study
Source: PLoS One. 2025 Oct 16;20(10):e0334740. doi: 10.1371/journal.pone.0334740 (PMC12530518; doi:10.1371/journal.pone.0334740)
Supplement: Attachment — Submitted filename: Response to Reviewers.docx [file pone.0334740.s007.docx]

**Response to reviewers**

We sincerely appreciate your time and valuable feedback on our manuscript. Below, we have addressed each of your comments in detail. Please note that the page and line numbers in our responses correspond to those in the clean version submitted as “Manuscript.”

**Editor**

Journal Requirements: When submitting your revision, we need you to address these additional requirements.

Comment: 1. Please ensure that your manuscript meets PLOS ONE's style requirements, including those for file naming.

**Response:** Thank you for your feedback. We have made efforts to ensure that our manuscript meets the style requirements of PLOS ONE.

Comment: 2. We note that your Data Availability Statement is currently as follows: All relevant data are within the manuscript and in Supporting Information files.

Please confirm at this time whether your submission contains all raw data required to replicate the results of your study. Authors must share the “minimal data set” for their submission. For example, authors should submit the following data:

- The values behind the means, standard deviations and other measures reported;

- The values used to build graphs;

- The points extracted from images for analysis.

Authors do not need to submit their entire data set if only a portion of the data was used in the reported study.

**Response:** Thank you for your observation regarding the Data Availability Statement. We attached minimal datasets for both the quantitative and qualitative study as supplementary files.

Comment: 3. When completing the data availability statement of the submission form, you indicated that you will make your data available on acceptance. We strongly recommend all authors decide on a data sharing plan before acceptance, as the process can be lengthy and hold up publication timelines. Please note that, though access restrictions are acceptable now, your entire data will need to be made freely accessible if your manuscript is accepted for publication. This policy applies to all data except where public deposition would breach compliance with the protocol approved by your research ethics board. If you are unable to adhere to our open data policy, please kindly revise your statement to explain your reasoning and we will seek the editor's input on an exemption. Please be assured that, once you have provided your new statement, the assessment of your exemption will not hold up the peer review process.

**Response:** Thank you for your feedback regarding sharing the data. We attached our minimal datasets for both the quantitative and qualitative study as supplementary files.

Comment: 4. Please include your full ethics statement in the ‘Methods’ section of your manuscript file. In your statement, please include the full name of the IRB or ethics committee who approved or waived your study, as well as whether or not you obtained informed written or verbal consent.

**Response:** Thank you for your comment. We have included our complete ethics statement, including the full name of the IRB and details about obtaining informed written consent or fingerprints, in the 'Methods' section (please refer to page 17, 361-370).

Reviewer 1

**Comment:** • The article is generally well-organized, with clear sections outlining the background, methodology, results, and discussion. Tables and figures are used effectively to present key findings (e.g., Tables 4 and 5 present a comprehensive breakdown of complementary feeding practices and factors associated with ACFP).

• However, the manuscript could benefit from more detailed descriptions of some of the statistical procedures, such as the generalized linear mixed model, as this might not be familiar to all readers.

**Response:** We are grateful for your thoughtful comment. We have tried to elaborate the generalized linear mixed model in detail to make it familiar to readers (kindly refer to page 14, lines 292-300).

**Comment:** The inclusion of more details on how the wealth index was calculated would enhance transparency in the methods.

**Response:** We have provided a detailed explanation of the wealth index calculation to enhance the transparency of our methods (please refer to pages 11 & 12, lines 240-257).

**Comment:** • The article generally adheres to standard reporting guidelines, including those for ethical conduct, informed consent, and transparency in data analysis.

However, further clarification on the data availability statement and adherence to data-sharing community standards (e.g., deposition in a public repository) could strengthen compliance with open data practices.

**Response:** Thank you for your suggestion. We attached minimal datasets for both the quantitative and qualitative study as supplementary files.

**Comment:** • The manuscript is written in clear, standard English with minimal grammatical errors. However, there are occasional awkward phrasings (e.g., "flesh foods" could be more clearly stated as "animal-source foods"). These minor issues do not significantly detract from the overall clarity but could be addressed to improve readability.

**Response:** We appreciate your suggestions for improving clarity. We have corrected grammatical errors throughout the manuscript. The phrase, “flesh foods” is used to specify the prevalence of meat consumption within the eight food groups considered for computing minimum dietary diversity. Using “animal source foods” may not clearly indicate this proportion, as “animal source foods’ include dairy products (milk, yogurt, and cheese), eggs, and flesh foods (please refer page 24, lines 456-460).

However, “animal-source foods” is used to describe whether mothers provided dairy products, eggs, or flesh foods to their children on fasting days (please refer page 42, lines 780-782).

**Comment:** • The manuscript could be improved by breaking up longer paragraphs, particularly in the discussion section, to enhance readability and clarity.

**Response:** Thank you for your feedback. We have improved the readability of the manuscript by breaking up long paragraphs in both the introduction and discussion sections.

Reviewer 2

**Comment:** **Study Population**

• The sentence in line 159 lacks clarity. Consider rephrasing for better readability.

**Response:** Thank you for your comment. The sentence in line 159 has been rephrased in the main document for clarity (page 8, line 159.

**Comment:** **Methods**

• If possible, include a flowchart to illustrate the steps involved in data collection.

**Response:** Thank you for your suggestion. Steps of data collection is illustrated using a flowchart for clarity. It is available as Fig 1 (kindly refer to page 11, lines 226-227).

**Comment:** **Independent Variables**

• List all the independent variables together.

**Response:** Thank you for your comment. All independent variables are listed together (please refer to page 11, line 232-238).

**Comment:** • Lines 381 and 396: Revise for clarity.

**Response:** Thank you for your recommendation. Both lines 381 and 396 have been corrected in the main document for clarity (please refer to pages 23, line 440-442) and page 24, lines 456-460).

**Comment:** **Results**

• If possible, use charts or plots to visually represent key findings of multivariate analysis.

**Response:** We appreciate your suggestion to enhance the visual representation of our multivariable analysis results. Currently, Table 5 presents the key multivariable analysis findings in a detailed format which includes all relevant statistical parameters (odds ratios, confidence intervals, and p-values). While we recognize the value of charts/plots for visualizing data, we found that the tabular format better preserves the precision needed for interpreting these key findings. However, we would be happy to add a supplementary figure if deemed essential (kindly refer to pages 28-231).

**Comment:** **Themes**

• Theme 1 and Theme 2: Review the statements from respondents for clarity. Some sentences require rewording to enhance readability and ensure accurate interpretation.

**Response:** Thank you for your feedback. Some words, phrases and sentences are reworded to enhance readability and ensure accurate interpretation for both theme 1 and 2 (please refer to pages 31-35).

**Comment:** **Discussion**

• When comparing findings related to a specific variable with previous research, discuss them within their context. Instead of using "This study" to refer to a result, consider specifying the exact figure or result for clarity. For example, lines 575 and 611–612 would benefit from such specificity.

**Response:** Thank you for your insightful suggestion. The phrase, “this study finding” (line 575) has been rephrased as “the 9.6% prevalence” (page 37, line 667). The phrase, “But this study's result” (611–612) has been rephrased as “However, this 13.7% prevalence” (page 39, line 711).

• “the present study's findings (page 32, line 569-570), has been rephrased as “this 9.6% prevalence” (page 37, line 662).

• This result (page 33, line 596), has been rephrased as “The 79.7% result” (page 38, line 695).

• This finding (page 34, line 600), rephrased as “This 79.7%” (page 38, line 699-700).

• This result (page 34, line 608), rephrased as “The prevalence of 13.7% MDD” (page 39, line 707).

**Comment:** • There are a few grammatical inconsistencies. A thorough proofreading of the manuscript is recommended to ensure clarity and coherence.

**Response:** Thank you for your feedback. Grammatical inconsistencies have been corrected throughout the manuscript to ensure clarity and coherence.

**Comment:** •Recommended course of action: Request Revision

**Response:** Thank you for your recommendation. We have revised the manuscript accordingly.

**Reviewer 3**

**Major Comments**

**Comment:** **1. Justification for Age Range (6–8 months)**

While the focus on infants aged 6–8 months aligns with WHO recommendations for the introduction of complementary foods, the authors should clarify why they did not extend the age group to 23 months. This limits comparability with other IYCF studies that use the full 6–23 months range. Further justification would strengthen the rationale.

**Response:** Thank you for your insightful feedback. Further justification has been added for why this study particularly focused on mothers with infants aged 6–8 months, rather than extending to 23 months (please refer to pages 4, lines 67-68 and page 6, lines 123-132).

**Comment:** **2. Definition of ACFP and Indicator Selection**

The use of revised 2021 WHO IYCF indicators, including breast milk as a food group, is commendable. However, the authors should explain the implications of this change on the comparability of their findings with studies using the 2008 guidelines.

**Response:** Thank you for highlighting this important aspect. The use of the revised WHO indicators (2021), other than the 2008 guidelines, emphasizes the inclusion of breastfeeding as one of the food groups in the MDD calculation. This change can affect MDD, MAD, and ACFP. In the current study, the prevalence of MDD was lower than study findings using the 2008 WHO IYCF practices indicators. This difference arises from the inclusion of breast milk as part of the eight food groups, compared to the calculation based on seven food groups. Evidence indicates that the MDD calculated based on the eight food group indicators is lower than the MDD-calculated using the seven food group indicators outlined in the 2008 WHO guidelines. Additionally, this study applied the new WHO guidelines to assess MDD, MAD, and ACFP, however, we referenced previous literature using the 2008 WHO guidelines in the discussion due to limited evidence supporting the new guidelines (we addressed this issue in the limitation section) (please refer to Pages 5, lines 89-94, page 39, lines 715-719 and page 44, lines 826-828).

**Comment:** • Additionally, the inclusion of egg/flesh food consumption as a separate indicator for ACFP needs further justification and clarity.

**Response:** Thank you for your feedback. Egg and flesh food consumption is one of the new complementary feeding indicators included in the revised WHO document for assessing IYCF practices. Eggs and meat are nutrient-dense food groups that are crucial for maximizing child nutrition and are vital for optimal linear growth (kindly refer to page 5, line 94-99).

**Comment:** **3. Low Prevalence of ACFP**

The reported prevalence of only 9.6% is alarmingly low. The discussion would benefit from a deeper exploration of its implications for public health, particularly in the context of national programs such as the Seqota Declaration and SDG targets.

**Response:** Thank you for your important observation. We have addressed the implications of the current very lower prevalence of ACFP in the context of practical and policy implications including the Ethiopia’s national program, the “Seqota Declaration of Zero Hunger”, which aims to “end stunting in children under two”, as well as the global commitment particularly the SDG target 2.2, which seeks to “end all forms of malnutrition by 2030” (please refer to pages 37 & 38, lines 675-681).

**Comment:** **4. Accounting for Cluster Design**

While a generalized linear mixed model is mentioned, the low intra-cluster correlation coefficient raises questions about the necessity of clustering in analysis. Please clarify how clustering was addressed statistically and whether simpler models were considered.

**Response:** Thank you for your insightful comment. Given the categorical nature of the outcome variable, a generalized linear mixed model (GLMM) was initially fitted to address cluster-level variables. The low intra-cluster correlation coefficient (ICC) indicates that most of the variance is within clusters rather than between them. However, we did not consider simpler models, such as generalized linear models (GLMs); instead, we used logistic regression (kindly refer to page 14, lines 292-298).

**Comment:** Also, explain the rationale behind selecting a design effect of 2.

**Response:** We multiplied the calculated sample size by a design effect of 2 to better account for the potential impact of clustering on estimates and to increase the overall sample size (please refer to page 14, lines 298-300).

**Comment:** **5. Measurement and Validation of Psychosocial Predictors**

The manuscript reports “perceived susceptibility” as a significant predictor of ACFP. More detail is needed on how this and other psychological constructs (severity, barriers, benefits) were measured and whether the instruments were validated in this population.

**Response:** Thank you for your valuable comment. The predisposing factors were assessed using a validated tool adapted, in accordance to child feeding practices, from a previous study conducted in the rural setting in Dessie, Amhara regional state, North central Ethiopia (kindly refer to pages 12 & 13, lines 2645-275).

**Comment:** **6. Integration of Mixed Methods**

Although both quantitative and qualitative components are well described, the manuscript would benefit from more explicit integration. A discussion section comparing, and contrasting quantitative findings with qualitative themes would improve cohesion.

**Response:** Thank you for your constructive feedback. We have tried to explicitly integrate the quantitative and qualitative findings in the discussion section to improve cohesion (Kindley refer pages 37, lines 657- 659; page 38, lines 683-685; page 39, lines 705-706; page 40, lines 742-747; page 41, lines 755-758 and 768-771; page 42, lines 782-788; page 42 & 43, lines 794-796; and page 43, lines 798-800).

**Comment:** **7. Wealth Index Construction**

The authors used principal component analysis (PCA) to construct a wealth index. Please provide additional detail: • Variables included in the PCA

**Response:** Thank you for your insightful comment. After performing rotations to make the components more interpretable, eleven variables with a commonality value greater than 0.5 were retained to produce factor scores. These variables include, households with 1) radio, 2) dining table, 3) sofa or chairs, 4) bed with cotton or sponge mattress, 5) cattle (cows or bulls), 6) horses, donkeys, or mules, 7) chickens, 8) watch (by at least one household member), 9) animal-drawn cart (by at least one household member), 10) location of drinking water, and 11) proximity of drinking water from home (please refer to page 12, lines 246-253).

**Comment:** • Percent variance explained by the first component

**Response:** The percent variance explained by the first component was 18.65%, whereas the first six components collectively explained a significant portion of the variance, which was 71.8% (kindly refer to page 12, line 253-254).

**Comment:** •Whether rotation was used

**Response:** In the PCA process, rotation was applied until all variables had a commonality value greater than 0.5 (kindly refer to page 12, line 246-248).

**Comment:** • Justification for dichotomizing variables and selecting quintiles

**Response:** Dichotomization of variables was used to simplify the analysis and facilitate the interpretation of findings. Wealth quintiles were employed to examine how equitably other indicators were distributed by wealth status (please refer to pages 12, lines 244-246 and page 12, lines 254-257).

**Comment:** • **8: Supporting Documents**

The manuscript mentions several supporting documents (e.g., protocol, SPIRIT checklist, ethical approvals, interview guide), but these were not accessible during review. Please ensure these materials are properly uploaded and available for editorial and reviewer access, especially given the study’s connection to a broader cluster RCT.

**Response:** Thank you for your observation. The required supplementary files, such as the Human Subjects Research Checklist, study protocol, interview guides (in English and Amharic), letter of ethical approval, quantitative minimal data set, and qualitative minimal data set have been uploaded as supporting information.

**Minor Comments**

**Comment:** **1. Language and Grammar**

The manuscript would benefit from professional language editing. Examples:

• Child feeding practices is a window..." should read “are a window…

• Age of infant [AOR=2.924, 95%CI: (1.992, 4.294)]” should be reworded for

clarity.

**Response:** Thank you for your insightful feedback.

• We have edited grammatical errors throughout the manuscript. • For example, the phrase “appropriate complementary feeding practices is” has been rephrased to “appropriate complementary feeding practices are.” (kindly refer to page 2, line 23).

• “Age of infant” has been rephrased to “A month increase in the age of the infant.” (please refer to page 2, line 37).

• We have revised and reordered the introduction section to streamline the focus, improve transitions between global, regional, and national contexts, and enhance the clarity and logical progression of ideas.

•We also removed sentences to avoid redundancy and for conciseness.

• We have also removed outdated findings and unnecessary details from the introduction and discussion sections.

**Comment:** **2. Ethical Considerations**

• While ethical approval is mentioned, the Methods section should explicitly state how informed consent was obtained from illiterate participants (e.g., fingerprint, witnessed verbal consent).

**Response:** Thank you for your important comment. We have explicitly state how informed consent was obtained from illiterate mothers (please refer to page 17, lines 365- 368).

**Comment:** **3. Figures and Tables**

• Figures 1 and 2 should be improved for clarity. Include titles, legends, and consistent color schemes. Consider placing key indicators (e.g., ACFP by age) into visually digestible formats.

**Response:** Thank you for your constructive feedback. We have tried to improve the clarity of figure 2 and 3 using TIFF figure format (Fig2: page 24, line 460-461 and Fig3: page 27, line 493-494).

**Comment:** **4. Limitations Section**

Please include a dedicated limitations section that acknowledges potential biases:

• Recall bias from 24-hour dietary recall

• Limited generalizability due to focus on rural areas

• Possible social desirability bias in qualitative interviews

**Response:** Thank you for your insightful comment. We have addressed the limitations of the study by acknowledging potential biases, including recall bias, generalizability issues, and social desirability bias. Additionally, we referenced previous literature using the 2008 WHO guidelines in the discussion due to the limited evidence supporting the new guidelines (kindly refer to page 44, lines 820-829).

**Comment:** **5. References**

• Ensure consistent formatting. Several references are outdated—please consider including more recent literature (2022–2023) on complementary feeding practices in LMICs for context.

**Response:** Thank you for your comment regarding the references. We have tried to use consistent formatting, removed outdated references, and used recent references.

**Comment:** **6. Conclusion and Recommendation**

• This is a relevant and well-conceived study with important implications for improving infant and young child feeding in Ethiopia. The mixed-methods approach is a strength, and the findings are robust. However, before acceptance, the authors should address the methodological clarifications, expand the integration of results, and improve the manuscript's readability and formatting.

**Response:** Thank you for your positive feedback on our study.

•We have addressed the methodological clarifications.

•We have tried to expand the integration of quantitative and qualitative findings in the discussion section.

•We have tried to improve the manuscript's readability and formatting throughout.

Additional revision

• We have revised the paragraph under the “Knowledge & attitudes toward ACFP” in the results section. The category names regarding mothers' knowledge of ACFP has been changed from high, medium, and low to good, average, and poor. Additionally, errors in frequency and percentage calculations have been corrected (Table 3) (kindly refer to pages 23, lines 440-442, and page 24, row 4).
