# Supplementary material for: Appropriate complementary feeding practices and associated factors among mothers with infants aged 6–8 months in West Gojjam Zone, Northwest Ethiopia: A mixed methods study
Source: PLoS One. 2025 Oct 16;20(10):e0334740. doi: 10.1371/journal.pone.0334740 (PMC12530518; doi:10.1371/journal.pone.0334740)
Supplement: Attachment — Submitted filename: Response_to_Reviewers_auresp_2.docx [file pone.0334740.s008.docx]

**Response to reviewers**

We sincerely appreciate your time and valuable feedback on our manuscript. This is our second revision, and we have thoughtfully addressed each of your comments in detail. Please note that the page and line numbers referenced in our responses correspond to those in the clean version submitted as “Manuscript.”

**Editor**

Journal Requirements:

**Comment:** Please review your reference list to ensure that it is complete and correct. If you have cited papers that have been retracted, please include the rationale for doing so in the manuscript text, or remove these references and replace them with relevant current references. Any changes to the reference list should be mentioned in the rebuttal letter that accompanies your revised manuscript. If you need to cite a retracted article, indicate the article’s retracted status in the References list and also include a citation and full reference for the retraction notice.

**Response:** Thank you for your comment regarding the reference list. We have carefully reviewed and revised the references list to ensure completeness and accuracy.

- In this revision, we removed duplicate references.
- We also added Digital Object Identifiers (DOIs) to make the references more complete.
- We have not cited any retracted articles, and the current version of the reference list contains no retracted publications. Thank you for highlighting this important aspect of manuscript quality.

**Reviewer 3**

**Comment:** Ensure figure legends and labels (especially for Figs 2 & 3) are clearly readable and interpretable without relying on the main text.

**Response:** Thank you for your feedback. We have carefully revised Figures 2 and 3 to ensure all percentages are accurate, legends, and labels are fully interpretable without reference to the main text. The specific improvements include:

**Figure 2 revisions:**

1. Added a clear, descriptive title,
2. Included “consumption categories” as a heading for Yes/No legends,
3. To ensure unambiguous interpretation, we explicitly defined 'Yes' as 'consumed' and 'No' as 'not consumed (kindly refer to Fig 2. (uploaded in our submission) and the figure caption on page 23, line 436).

**Figure 3 revisions:**

1. Incorporated a clear, descriptive title,
2. Included “Categories and sample sizes by age” as a heading for age classifications (6m, 7m, and 8m), along with the corresponding population size for each age group,
3. Calculation errors were corrected for child feeding indicators, including Minimum meal frequency, Minimum acceptable diet, Egg and/or flesh food consumption, Appropriate complementary feeding practices,
4. Replaced all abbreviations of indicators with full terms to enhance clarity (kindly refer Fig 3. (uploaded in our submission) and the figure caption on page 26, lines 469-470).

These improvements may ensure both figures now contain all necessary information for standalone interpretation.

**Comment:** Consider adding a brief, summarizing paragraph at the end of the discussion explicitly comparing quantitative and qualitative findings.

**Response:** Thank you for your valuable suggestion. We have added a brief summarizing paragraph at the end of the discussion section comparing the quantitative and qualitative findings (please refer to pages 41 and 42, lines 764-773).

Additional revision

We have carefully reviewed and revised the manuscript throughout to address:

- Grammatical and spelling errors, unnecessary spacing, and punctuation issues,
- Consistency in the use of abbreviations,
- Redundant or unclear sentences, to improve clarity, consistency/ flow, and readability.

**In the qualitative section:**

- Some quotes were conceptually shortened to enhance clarity and coherence while preserving their original meaning.
- To minimize the risk of identifying participants, quote attributions (speaker tags) were rephrased or removed.
