# Supplementary material for: Appropriate complementary feeding practices and associated factors among mothers with infants aged 6–8 months in West Gojjam Zone, Northwest Ethiopia: A mixed methods study
Source: PLoS One. 2025 Oct 16;20(10):e0334740. doi: 10.1371/journal.pone.0334740 (PMC12530518; doi:10.1371/journal.pone.0334740)
Supplement: Attachment — Submitted filename: Reviewer Comments for Manuscript.pdf [file pone.0334740.s005.pdf]

Reviewer Comments for Manuscript: PONE-D-24-46708

Title: *Appropriate complementary feeding practices and associated factors among mothers with infants aged 6–8 months in the West Gojjam Zone, Northwest Ethiopia: A mixed methods study*
Journal: PLOS ONE

General Assessment

This manuscript presents a mixed-methods study investigating the prevalence and associated factors of appropriate complementary feeding practices (ACFP) among mothers with infants aged 6–8 months in the West Gojjam Zone, Ethiopia. The combination of quantitative and qualitative data is a strength, allowing for both breadth and depth in understanding the drivers and barriers to optimal infant feeding practices. The study is relevant to public health practitioners and policymakers seeking to improve child nutrition outcomes in resource-limited settings. However, several areas require clarification, methodological refinement, and editorial improvement before the manuscript is ready for publication.

Major Comments

1. **Justification for Age Range (6–8 months)**

   While the focus on infants aged 6–8 months aligns with WHO recommendations for the introduction of complementary foods, the authors should clarify why they did not extend the age group to 23 months. This limits comparability with other IYCF studies that use the full 6–23 months range. Further justification would strengthen the rationale.

2. **Definition of ACFP and Indicator Selection**

   The use of revised 2021 WHO IYCF indicators, including breast milk as a food group, is commendable. However, the authors should explain the implications of this change on the comparability of their findings with studies using the 2008 guidelines. Additionally, the inclusion of egg/flesh food consumption as a separate indicator for ACFP needs further justification and clarity.

3. **Low Prevalence of ACFP**

The reported prevalence of only 9.6% is alarmingly low. The discussion would benefit from a deeper exploration of its implications for public health, particularly in the context of national programs such as the Seqota Declaration and SDG targets.

4. **Accounting for Cluster Design**

While a generalized linear mixed model is mentioned, the low intra-cluster correlation coefficient raises questions about the necessity of clustering in analysis. Please clarify how clustering was addressed statistically and whether simpler models were considered. Also, explain the rationale behind selecting a design effect of 2.

5. **Measurement and Validation of Psychosocial Predictors**

The manuscript reports "perceived susceptibility" as a significant predictor of ACFP. More detail is needed on how this and other psychological constructs (severity, barriers, benefits) were measured and whether the instruments were validated in this population.

6. **Integration of Mixed Methods**

Although both quantitative and qualitative components are well described, the manuscript would benefit from more explicit integration. A discussion section comparing and contrasting quantitative findings with qualitative themes would improve cohesion.

7. **Wealth Index Construction**

The authors used principal component analysis (PCA) to construct a wealth index. Please provide additional detail:

- Variables included in the PCA
- Percent variance explained by the first component
- Whether rotation was used
- Justification for dichotomizing variables and selecting quintiles

8. **Supporting Documents**

The manuscript mentions several supporting documents (e.g., protocol, SPIRIT checklist, ethical approvals, interview guide), but these were not accessible during review. Please ensure these materials are properly uploaded and available for editorial and reviewer access, especially given the study's connection to a broader cluster RCT.

9.

Minor Comments

1. **Language and Grammar**

The manuscript would benefit from professional language editing. Examples:

- "Child feeding practices is a window..." should read "are a window…"
- "Age of infant [AOR=2.924, 95%CI: (1.992, 4.294)]" should be reworded for clarity.

2. **Ethical Considerations**

While ethical approval is mentioned, the Methods section should explicitly state how informed consent was obtained from illiterate participants (e.g., fingerprint, witnessed verbal consent).

3. **Figures and Tables**

Figures 1 and 2 should be improved for clarity. Include titles, legends, and consistent color schemes. Consider placing key indicators (e.g., ACFP by age) into visually digestible formats.

4. **Limitations Section**

Please include a dedicated limitations section that acknowledges potential biases:

- Recall bias from 24-hour dietary recall
- Limited generalizability due to focus on rural areas
- Possible social desirability bias in qualitative interviews

5. **References**
   Ensure consistent formatting. Several references are outdated—please consider including more recent literature (2022–2023) on complementary feeding practices in LMICs for context.

Conclusion and Recommendation

This is a relevant and well-conceived study with important implications for improving infant and young child feeding in Ethiopia. The mixed-methods approach is a strength, and the findings are robust. However, before acceptance, the authors should address the methodological clarifications, expand the integration of results, and improve the manuscript's readability and formatting.
